# Design, synthesis and anticancer activity of naphthoquinone derivatives

**DOI:** 10.1080/14756366.2020.1740693

**Published:** 2020-03-23

**Authors:** Xiao-bao Shen, Yang Wang, Xuan-zhen Han, Liang-quan Sheng, Fu-fang Wu, Xinhua Liu

**Affiliations:** a School of Pharmacy, Anhui Medical University, Hefei, PR China; b Engineering Research Center of Biomass Conversion and Pollution Prevention of Anhui Educational Institutions, Fuyang Normal University, Fuyang, PR China

**Keywords:** Naphthoquinone moiety, anticancer activity, autophagy

## Abstract

Basis on molecular docking and pharmacophore analysis of naphthoquinone moiety, a total of 23 compounds were designed and synthesised. With the help of reverse targets searching, anti-cancer activity was preliminarily evaluated, most of them are effective against some tumour cells, especially compound **12**: 1-(5,8-dihydroxy-1,4-dioxo-1,4-dihydronaphthalen-2-yl)-4-methylpent-3-en-1-yl-4-oxo-4-((4-phenoxyphenyl)amino) butanoate whose IC_50_ against SGC-7901 was 4.1 ± 2.6 μM. Meanwhile the anticancer mechanism of compound **12** had been investigated by AnnexinV/PI staining, immunofluorescence, Western blot assay and molecular docking. The results indicated that this compound might induce cell apoptosis and cell autophagy through regulating the PI3K signal pathway.

## 1. Introduction

Gastric cancer (GC) is a severe malignant tumour associated with high mortality, especially in Asia^1^^,^^2^. Due to the non-specific symptoms in early appearance and the poor prognosis, the 5-year relative survival rate for GC was at most 20%^3^. Because of GC difficult diagnosis and chemotherapy resistance, it is necessary to investigate the new therapeutic drugs and deeply explore its anti-tumour mechanism^4^.

Autophagy is a pathway participated in the degradation of lysosomal, which contributed to renew the needed energy of cell survival during starvation^5^^,^^6^ and plays an important role in GC. Autophagy is expected to be a new target for molecular therapy^7^ which can inhibit the generation of tumour, on the other hand, can promote the survival and transfer of tumour cells^8^. A large number of studies have shown that autophagy is closely related to DC. For example, the gene polymorphism of autophagyis related to the susceptibility of GC^9^^,^^10^. Since lots of compounds regulating autophagy are discovered, but, the tumour microenvironments are complexity, the role of it in the tumour cells is not very clear. Therefore, it is necessary to reveal the title compounds’ anticancer mechanism through rational regulating of autophagy.

Compounds with naphthoquinone moiety show good activity against breast cancer, liver cancer, human cervical carcinoma and GC^11–14^ through inducing cell apoptosis^15–21^. Inducing autophagy is also one of the main mechanisms for anti-cancer activity^13^^,^^22^^,^^23^. Among them, PI3K and mTOR signalling pathways have been proved to be the main signal pathways to regulate autophagy^24–27^. However, for these kinds of derivatives, the poor selectivity and high cytotoxicity limit their clinical application. Therefore, how to reduce the toxicity and increase the selectivity is the main trend. Because the hydroxyl group of naphthoquinone is easily oxidised, and the polyhydroxy is not conducive to selectivity, so modification of the hydroxyl group is one of the main methods to study structure–activity relationship. Many reports showed that the introduction of suitable substituents on the hydroxyl group could improve activity^28–30^ and reduce toxicity^31^. Furthermore, Ahn^32^ and Sankawa^33^ showed that the side chain hydroxyl was not essential for activity. Both acylation and etherification could maintain antitumor activity at low concentration and the introduction of double bond should increase the activity obviously.

**Figure 1.**
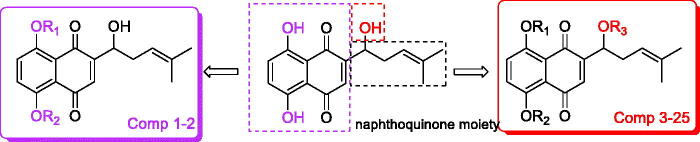
The general design strategy in this study.

So, based on the above, combined with the results of reverse target finding, a series of new naphthoquinone-esterification/etherification derivatives targeting PI3K were designed and synthesised (Figure 1). Their inhibitory activities against SGC-7901, MGC-803, SMMC-7721, and U-87 cells were determined by MTT assay. The molecular mechanism of title compound was studied by influencing the signal pathway of PI3K/AKT/mTOR. Meanwhile molecular docking was used to determine the interaction between the compound and PI3K protein.

## 2. Experimental section

### 2.1. Chemistry

Adriamycin was purchased from Aladdin Chemical Reagent Co., Ltd (Shanghai, China) and other reagents were purchased from Abcam, Cell Signalling Technology (Boston, MA) and Beyotime (Shanghai, China). ^1^H NMR spectra were recorded on 400 MHz or 600 MHz and ^13 ^C NMR spectra were obtained at 100 MHz or 150 MHz (Supplementary material).

#### 2.1.1. General procedure for preparation of compounds 1 and 2

To a solution of shikonin in DMF was added iodomethane (CH_3_I, 2.0 equiv) orbenzyl bromide and K_2_CO_3_ (2.0 equiv) and the mixture was stirred at 60 °C for 6 h. The mixture was filtered and the residue was washed with EA, the filtrate was extracted by EA and combined organic layers were washed with water and brine. Evaporate the solvent and the products were purified by column chromatography to give product as a solid.

**1**: *2-(1-hydroxy-4-methylpent-3-en-1-yl)-5,8-dimethoxynaphthalene-1,4-dione,* Brown solid. Yield: 92.3%. m.p. 94.3 °C. 1H NMR (600 MHz, CDCl_3_) *δ* = 7.30 (s, 2H), 6.79 (s, 1H), 5.18–5.13 (m, 1H), 4.77–4.72 (m, 1H), 3.95 (s, 6H), 2.62–2.54 (m, 1H), 2.50 (s, 1H), 2.39–2.33 (m, J = 14.7, 7.5, 1H), 1.71 (s, 3H), 1.61 (s, 32H).

**2:** *5,8-bis(benzyloxy)-2-(1-hydroxy-4-methylpent-3-en-1-yl)naphthalene-1,4-dione,* Deep red solid. Yield: 37.6%. m.p. 60.0 °C.^1^H NMR (600 MHz, CDCl_3_) *δ* = 12.57 (s, 1H), 12.42 (s, 1H), 7.31–7.17 (m, 7H), 6.86 (s, 1H), 6.02 (dd, *J* = 6.7, 4.9, 1H), 5.06 (t, *J* = 7.2, 1H), 2.97 (t, *J* = 7.7, 2H), 2.79–2.67 (m, 2H), 2.62–2.54 (m, 1H), 2.48–2.42 (m, 1H), 1.67 (s, 3H), 1.56 (s, 3H). ^13 ^C NMR (150 MHz, CDCl_3_) *δ* 178.18, 176.68, 171.69, 167.39, 166.87, 148.11, 140.04, 136.08, 132.85, 132.68, 131.46, 128.56, 128.21, 126.43, 117.62, 111.78, 111.53, 69.48, 35.75, 32.83, 30.80, 25.76, 17.95. HR-ESI-MS: *m*/*z* [M + H]+ calcd for 469.2064

#### 2.1.2. General procedure for preparation of compounds 3–24

To a solution of amine (1 equiv) in toluene (dichloromethane or furanidine according the amine’s solubility) was added succinic anhydride (1.1 equiv) or maleic anhydride (1.1 equiv). Then the solution was refluxed for 0.5 h. After TLC shows the reaction was completed, the solvent was cooled to room temperature. After filtration, cooled toluene was used washing the precipitate to give the crude product, if the product was dissolved in the solvent, the solvent was then removed under reduced pressure to crude product for next step directly.

At 0 °C, dicyclohexyl carbodiimide and 4-dimethylaminopyridine (DMAP) were added and stirred in the solution of above-mentioned carboxylic acid and dichloromethane (DCM) for about 15 min. Then solution of shikonin in DCM was dropped, the combined solution was stirred in ice bath for 6 h, slowly to room temperature. After TLC showed the reaction was completed, the solution was concentrated and cooled to −10 °C and filtered to removal most of the dicyclohexylurea (DCU), the filtrate was evaporated, the product was purified via prepared TLC to give deep red solid.

**3**: *1-(5,8-dihydroxy-1,4-dioxo-1,4-dihydronaphthalen-2-yl)-4-methylpent-3-en-1-yl-3-phenylpropanoate,* deep red solid. Yield: 37.6%. m.p. 60.0 °C. ^1^H NMR (600 MHz, CDCl_3_) *δ* = 12.57 (s, 1H), 12.42 (s, 1H), 7.31–7.17 (m, 7H), 6.86 (s, 1H), 6.02 (dd, *J* = 6.7, 4.9, 1H), 5.06 (t, *J* = 7.2, 1H), 2.97 (t, *J* = 7.7, 2H), 2.79–2.67 (m, 2H), 2.62–2.54 (m, 1H), 2.48–2.42 (m, 1H), 1.67 (s, 3H), 1.56 (s, 3H). ^13 ^C NMR (150 MHz, CDCl_3_) *δ* 178.18, 176.68, 171.69, 167.39, 166.87, 148.11, 140.04, 136.08, 132.85, 132.68, 131.46, 128.56, 128.21, 126.43, 117.62, 111.78, 111.53, 69.48, 35.75, 32.83, 30.80, 25.76, 17.95. HR-ESI-MS: *m*/*z* [M + H]^+^ calcd for 421.1632.

**4**: *1-(5,8-dihydroxy-1,4-dioxo-1,4-dihydronaphthalen-2-yl)-4-methylpent-3-en-1-yl cinnamate,* red solid. Yield: 64.6%. m.p. 85.0 °C. ^1^H NMR (600 MHz, CDCl_3_) *δ* = 12.61 (s, 1H), 12.50 (s, 1H), 7.75 (d, *J* = 16.3, 1H), 7.73–7.70 (m, 1H), 7.54–7.50 (m, 2H), 7.44–7.39 (m, 2H), 7.21–7.16 (m, 3H), 6.45 (d, *J* = 16.2, 1H), 5.23–5.19 (m, 1H), 4.94–4.91 (m, 1H), 2.68–2.62 (m, 1H), 2.39–2.33 (m, 1H), 1.76 (s, 3H), 1.66 (s, 3H). ^13 ^C NMR (150 MHz, CDCl_3_) *δ* 180.74, 179.95, 167.72, 165.38, 164.77, 153.99, 151.40, 137.52, 132.42, 132.27, 131.83, 130.91, 128.81, 128.26, 118.39, 112.02, 111.54, 103.67, 65.57, 35.67, 25.97, 18.09. HR-ESI-MS: *m*/*z* [M + H]^+^ calcd for 419.1482.

**5**: *1-(5,8-dihydroxy-1,4-dioxo-1,4-dihydronaphthalen-2-yl)-4-methylpent-3-en-1-yl (E)-4-(3,4,5-trimethoxyphenyl)but-3-enoate,* red solid. Yield: 42.5%. m.p. 54.0 °C. ^1^H NMR (600 MHz, CDCl_3_) *δ* = 12.60 (s, 1H), 12.41 (s, 1H), 7.62 (d, *J* = 15.9, 1H), 7.17 (s, 2H), 7.04 (s, 1H), 6.76 (s, 2H), 6.39 (d, *J* = 15.9, 1H), 6.14–6.11 (m, 1H), 5.16 (t, *J* = 6.8, 1H), 3.89 (s, 6H), 3.87 (s, 3H), 1.67 (s, 3H), 1.57 (s, 3H). 13 C NMR (150 MHz, CDCl_3_) *δ* 178.16, 176.67, 167.51, 166.98, 165.60, 153.42, 148.24, 145.89, 140.33, 136.08, 132.88, 132.74, 131.50, 129.56, 117.69, 116.45, 111.82, 111.56, 105.33, 69.60, 60.96, 56.16, 32.90, 25.77, 18.00, 11.20. HR-ESI-MS: *m*/*z* [M + H]^+^ calcd for 523.1964.

**6**: *1-(5,8-dihydroxy-1,4-dioxo-1,4-dihydronaphthalen-2-yl)-4-methylpent-3-en-1-yl-2-(4-chlorophenoxy)acetate,* deep red solid. Yield: 85.2%. m.p. 102.0 °C. ^1^H NMR (600 MHz, CDCl_3_) *δ* = 12.57 (s, 1H), 12.39 (s, 1H), 7.26–7.22 (m, *J* = 10.1, 2H), 7.19–7.16 (m, 2H), 6.93 (s, 1H), 6.84 (d, *J* = 8.9, 2H), 6.15 (dd, *J* = 7.2, 4.7, 1H), 5.03 (t, *J* = 7.2, 1H), 4.68 (s, 2H), 2.66–2.60 (m, 1H), 2.51–2.47 (m, 1H), 1.67 (s, 3H), 1.56 (s, 3H). ^13 ^C NMR (150 MHz, CDCl_3_) *δ* 176.20, 174.62, 169.24, 168.73, 167.64, 156.20, 146.63, 136.59, 133.56, 133.35, 131.17, 129.54, 126.93, 117.14, 115.86, 111.75, 111.53, 70.53, 65.40, 32.76, 25.76, 17.95. HR-ESI-MS: *m*/*z* [M + H]^+^ calcd for 457.1032.

**7**: *1-(5,8-dihydroxy-1,4-dioxo-1,4-dihydronaphthalen-2-yl)-4-methylpent-3-en-1-yl-4-oxo-4-(piperidin-1-yl)butanoate,* red solid. Yield: 65.2%. m.p. 120.0 °C.^1^H NMR (600 MHz, CDCl_3_) *δ* = 12.56 (s, 1H), 12.43 (s, 1H), 7.19–7.14 (m, 2H), 7.06 (s, 1H), 6.03–5.98 (m, 1H), 5.16-5.08 (m, 1H), 3.57–3.47 (m, 2H), 3.42–3.33 (m, 2H), 2.73 (t, *J* = 6.4, 2H), 2.66–2.57 (m, *J* = 18.5, 11.9, 3H), 2.52–2.44 (m, 1H), 1.67 (s, 3H), 1.64–1.60 (m, *J* = 4.3, 2H), 1.57–1.48 (m, 7H). ^13 ^C NMR (150 MHz, CDCl_3_) *δ* 194.47, 178.95, 177.49, 172.20, 168.93, 166.70, 166.18, 148.19, 135.96, 132.58, 132.35, 131.97, 117.71, 111.57, 69.62, 65.57, 46.29, 42.87, 32.77, 29.41, 27.92, 26.32, 25.77, 24.48, 17.96. HR-ESI-MS: *m*/*z* [M + H]^+^ calcd for 456.2042.

**8**: *1-(5,8-dihydroxy-1,4-dioxo-1,4-dihydronaphthalen-2-yl)-4-methylpent-3-en-1-yl 4-morpholino-4-oxobutanoate,* red solid. Yield: 26.4%. m.p. 105.0 °C.^1^H NMR (600 MHz, CDCl_3_) *δ* = 12.56 (s, 1H), 12.43 (s, 1H), 7.19–7.14 (m, 2H), 7.05 (s, 1H), 6.01 (dd, *J* = 6.3, 5.3, 1H), 5.12 (t, *J* = 7.2, 1H), 3.68–3.63 (m, 4H), 3.61–3.58 (m, 2H), 3.48–3.45 (m, 2H), 2.75 (t, J = 6.6, 2H), 2.67–2.58 (m, 3H), 2.53–2.43 (m, 1H), 1.68 (s, 3H), 1.56 (s, 3H). ^13 ^C NMR (150 MHz, CDCl_3_) *δ* 178.62, 177.13, 172.00, 169.50, 167.02, 166.50, 148.01, 136.03, 132.71, 132.49, 131.85, 117.65, 111.80, 111.56, 69.77, 66.83, 66.46, 45.61, 42.06, 32.76, 29.17, 27.71, 25.78, 17.96. HR-ESI-MS: *m*/*z* [M + H]^+^ calcd for 458.1831.

**9**: *1-(5,8-dihydroxy-1,4-dioxo-1,4-dihydronaphthalen-2-yl)-4-methylpent-3-en-1-yl-4-(3,4-dihydroquinolin-1(2H)-yl)-4-oxobutanoate*, red solid. Yield: 44.1%. m.p. 92.0 °C. ^1^H NMR (600 MHz, CDCl_3_) *δ* = 12.57 (s, 1H), 12.44 (s, 1H), 7.19–7.07 (m, 6H), 7.05 (s, 1H), 6.02–5.98 (m, 1H), 5.11 (t, *J* = 7.0, 1H), 3.83–3.73 (m, 2H), 2.82–2.78 (m, 2H), 2.77–2.75 (m, 2H), 2.72–2.69 (m, 2H), 2.64–2.58 (m, 1H), 2.52–2.45 (m, 1H), 1.98–1.88 (m, 2H), 1.67 (s, 3H), 1.55 (s, 3H). ^13 ^C NMR (150 MHz, CDCl_3_) *δ* 178.72, 177.25, 171.93, 166.89, 166.38, 156.74, 148.11, 136.00, 132.65, 132.43, 131.88, 128.54, 126.10, 124.68, 117.66, 111.80, 111.56, 69.69, 49.12, 33.92, 32.77, 29.62, 26.77, 25.76, 25.59, 24.93, 23.96, 17.95. HR-ESI-MS: *m*/*z* [M + H]^+^ calcd for 504.2024.

**10**: *1-(5,8-dihydroxy-1,4-dioxo-1,4-dihydronaphthalen-2-yl)-4-methylpent-3-en-1-yl-4-((4-methoxyphenyl)amino)-4-oxobutanoate,* red solid. Yield: 72.9%. m.p. 115.0 °C. ^1^H NMR (600 MHz, CDCl_3_) *δ* = 12.57 (s, 1H), 12.41 (s, 1H), 7.36 (d, *J* = 8.8, 2H), 7.34 (s, 1H), 7.19–7.15 (m, 2H), 7.04 (s, 1H), 6.82 (d, *J* = 8.8, 2H), 6.04 (dd, *J* = 6.4, 5.3, 1H), 5.11 (t, *J* = 7.0, 1H), 3.77 (s, 3H), 2.83 (t, *J* = 6.6, 2H), 2.65 (t, *J* = 6.5, 2H), 2.62–2.58 (m, 1H), 2.52–2.46 (m, 1H), 1.66 (s, 3H), 1.56 (s, 3H). ^13 ^C NMR (150 MHz, CDCl_3_) *δ* 207.18, 177.88, 176.40, 172.17, 169.21, 168.00, 167.47, 156.52, 147.82, 136.41, 133.16, 132.93, 131.79, 130.96, 121.70, 117.65, 114.23, 111.96, 111.72, 70.15, 55.60, 32.96, 31.98, 31.09, 29.73, 25.90, 18.11. HR-ESI-MS: *m*/*z* [M + H]^+^ calcd for 494.1839.

**11**: *1-(5,8-dihydroxy-1,4-dioxo-1,4-dihydronaphthalen-2-yl)-4-methylpent-3-en-1-yl-4-((4-fluoro-3-(trifluoromethyl)phenyl)amino)-4-oxobutanoate*, red solid. Yield: 63.2%. m.p. 137.0 °C. ^1^H NMR (600 MHz, CDCl_3_) *δ* = 12.56 (s, 1H), 12.39 (s, 1H), 7.76–7.72 (m, 1H), 7.70–7.63 (m, 2H), 7.19–7.13 (m, 2H), 7.11 (t, *J* = 9.3, 1H), 7.03 (s, 1H), 6.07–6.02 (m, 1H), 5.10 (t, *J* = 6.9, 1H), 2.85–2.81 (m, 2H), 2.69–2.58 (m, 3H), 2.52–2.45 (m, 1H), 1.65 (s, 3H), 1.56 (s, 3H). ^13 ^C NMR (150 MHz, CDCl_3_) *δ* 207.09, 177.19, 175.67, 172.03, 169.54, 168.32, 167.80, 147.36, 136.32, 136.29, 133.89, 133.20, 132.96, 131.44, 124.85, 118.30, 117.39, 117.22, 111.76, 111.51, 70.21, 32.78, 31.78, 30.92, 29.30, 25.71, 17.93. HR-ESI-MS: *m*/*z* [M + H]^+^ calcd for 550.1437.

**12**: *1-(5,8-dihydroxy-1,4-dioxo-1,4-dihydronaphthalen-2-yl)-4-methylpent-3-en-1-yl-4-oxo-4-((4-phenoxyphenyl)amino)butanoate,* red solid. Yield: 82.1%. m.p. 133.0 °C. ^1^H NMR (600 MHz, CDCl_3_) *δ* = 12.59–12.55 (m, 1H), 12.40 (s, 1H), 7.47–7.38 (m, 3H), 7.30 (t, *J* = 7.8, 2H), 7.19–7.13 (m, 2H), 7.07 (t, *J* = 7.4, 1H), 7.04 (s, 1H), 6.95 (t, *J* = 9.5, 4H), 6.06–6.02 (m, 1H), 5.10 (t, *J* = 7.2, 1H), 2.84 (t, *J* = 6.5, 2H), 2.66 (t, *J* = 6.5, 2H), 2.64–2.57 (m, 1H), 2.49–2.42 (m, 1H), 1.66 (s, 3H), 1.56 (s, 3H). ^13 ^C NMR (150 MHz, CDCl_3_) *δ* 177.60, 176.09, 172.01, 169.20, 167.94, 167.42, 153.39, 147.59, 136.26, 133.21, 133.05, 132.82, 131.57, 129.68, 123.02, 121.42, 119.57, 118.36, 117.46, 111.78, 111.55, 70.04, 32.80, 31.83, 29.48, 25.73, 17.95. HR-ESI-MS: *m*/*z* [M + H]^+^ calcd for 556.1958.

**13**: *1-(5,8-dihydroxy-1,4-dioxo-1,4-dihydronaphthalen-2-yl)-4-methylpent-3-en-1-yl-4-oxo-4-((4-(trifluoromethoxy)phenyl)amino)butanoate*, red solid. Yield: 58.6%. m.p. 134.0 °C. ^1^H NMR (600 MHz, CDCl_3_) *δ* = 12.56 (s, 1H), 12.40 (s, 1H), 7.56 (br, 1H), 7.50 (d, *J* = 8.7, 2H), 7.18–7.11 (m, 4H), 7.04 (s, 1H), 6.05–6.02 (m, 1H), 5.10 (t, *J* = 7.0, 1H), 2.83 (t, *J* = 6.4, 2H), 2.67 (t, *J* = 6.3, 2H), 2.64–2.58 (m, 1H), 2.52–2.45 (m, 1H), 1.65 (s, 3H), 1.55 (s, 3H). ^13 ^C NMR (150 MHz, CDCl_3_) *δ* 177.38, 175.87, 171.99, 169.27, 168.15, 167.62, 147.45, 136.75, 136.29, 133.14, 132.89, 131.88, 131.53, 121.13, 117.42, 116.79, 111.76, 111.54, 70.12, 32.78, 32.00, 30.92, 29.41, 25.73, 17.94. HR-ESI-MS: *m*/*z* [M + H]^+^ calcd for 548.1546.

**14**: *1-(5,8-dihydroxy-1,4-dioxo-1,4-dihydronaphthalen-2-yl)-4-methylpent-3-en-1-yl-4-((4-bromophenyl)amino)-4-oxobutanoate,* red solid. Yield: 77.6%. m.p. 105.0 °C. ^1^H NMR (600 MHz, CDCl_3_) *δ* = 12.56 (s, 1H), 12.40 (s, 1H), 7.47 (br, 1H), 7.40–7.33 (m, 4H), 7.19–7.14 (m, 2H), 7.03 (s, 1H), 6.06–6.01 (m, 1H), 5.09 (t, *J* = 7.2, 1H), 2.82 (t, *J* = 6.4, 2H), 2.68–2.57 (m, 3H), 2.53–2.44 (m, 1H), 1.65 (s, 3H), 1.55 (s, 3H). ^13 ^C NMR (150 MHz, CDCl_3_) *δ* 177.38, 175.87, 171.99, 169.27, 168.15, 167.62, 147.45, 136.75, 136.29, 133.14, 132.89, 131.88, 131.53, 121.13, 117.42, 116.79, 111.76, 111.54, 70.12, 32.78, 32.00, 30.92, 29.41, 25.73, 17.94. HR-ESI-MS: *m*/*z* [M + H]^+^ calcd for 542.0732.

**15**: *1-(5,8-dihydroxy-1,4-dioxo-1,4-dihydronaphthalen-2-yl)-4-methylpent-3-en-1-yl-4-((2-methoxyphenyl)amino)-4-oxobutanoate,* red solid. Yield: 87.3%. m.p. 113.0 °C. ^1^H NMR (600 MHz, CDCl_3_) *δ* = 12.57 (s, 1H), 12.42 (s, 1H), 8.32 (d, *J* = 7.8, 1H), 7.85 (br, 1H), 7.18–7.14 (m, 2H), 7.05–7.04 (m, 1H), 7.02–6.98 (m, 1H), 6.95–6.91 (m, 1H), 6.83 (d, *J* = 8.2, 1H), 6.05–6.02 (m, 1H), 5.13–5.08 (m, 1H), 3.85 (s, 3H), 2.83 (t, *J* = 6.6, 2H), 2.72 (t, *J* = 6.5, 2H), 2.64–2.57 (m, 1H), 2.51–2.45 (m, 1H), 1.66 (s, 3H), 1.55 (s, 3H). ^13 ^C NMR (150 MHz, CDCl_3_) *δ* 207.02, 177.72, 176.24, 172.01, 169.05, 167.84, 167.31, 156.36, 147.66, 136.25, 133.00, 132.77, 131.63, 130.80, 121.54, 117.49, 114.07, 111.74, 111.56, 69.99, 55.44, 32.80, 31.82, 30.93, 29.57, 25.74, 17.95. HR-ESI-MS: *m*/*z* [M + H]^+^ calcd for 494.1853.

**16**: *1-(5,8-dihydroxy-1,4-dioxo-1,4-dihydronaphthalen-2-yl)-4-methylpent-3-en-1-yl-4-(naphthalen-1-ylamino)-4-oxobutanoate,* red solid. Yield: 74.8%. m.p. 127.0 °C.^1^H NMR (600 MHz, CDCl_3_) *δ* = 12.54 (s, 1H), 12.26 (s, 1H), 7.94 (d, *J* = 7.1, 1H), 7.89 (br, 1H), 7.82–7.76 (m, 2H), 7.64 (d, *J* = 8.2, 1H), 7.47–7.37 (m, 3H), 7.17–7.10 (m, 2H), 6.97 (s, 1H), 6.11–6.04 (m, 1H), 5.14–5.05 (m, 1H), 2.93 (t, *J* = 5.5, 2H), 2.87–2.79 (m, 1H), 2.63–2.57 (m, 2H), 2.51–2.43 (m, 1H), 1.63 (s, 3H), 1.54 (s, 3H). ^13 ^C NMR (150 MHz, CDCl_3_) *δ* 177.20, 172.38, 169.95, 167.43, 156.69, 147.43, 136.30, 133.90, 133.00, 132.72, 132.06, 131.41, 130.89, 128.81, 128.65, 126.66, 126.15, 125.85, 125.70, 125.63, 120.45, 117.43, 111.73, 70.18, 33.92, 32.77, 25.58, 24.91, 17.93. HR-ESI-MS: *m*/*z* [M + H]^+^ calcd for 514.1846.

**17**: *1-(5,8-dihydroxy-1,4-dioxo-1,4-dihydronaphthalen-2-yl)-4-methylpent-3-en-1-yl-4-((3-chlorophenethyl)amino)-4-oxobutanoate,* red solid. Yield: 46.3%. m.p. 91.8 °C. ^1^H NMR (600 MHz, CDCl_3_) *δ* = 12.57 (s, 1H), 12.41 (s, 1H), 7.23–7.14 (m, 5H), 7.05 (d, *J* = 7.2, 1H), 7.00 (s, 1H), 6.01 (dd, *J* = 6.3, 5.1, 1H), 5.61 (s, 1H), 5.10 (t, *J* = 7.3, 1H), 3.49–3.42 (m, 2H), 2.77–2.72 (m, 4H), 2.62–2.54 (m, 1H), 2.48–2.38 (m, 3H), 1.67 (s, 3H), 1.56 (s, 3H). ^13 ^C NMR (150 MHz, CDCl_3_) *δ* 177.84, 176.33, 171.85, 171.02, 167.74, 167.20, 147.82, 140.82, 136.17, 134.34, 132.97, 132.74, 131.54, 129.84, 128.85, 126.91, 126.71, 117.53, 111.78, 111.54, 69.86, 40.53, 35.29, 32.81, 30.81, 29.44, 25.76, 17.95. HR-ESI-MS: *m*/*z* [M + H]^+^ calcd for 526.1632.

**18**: *1-(5,8-dihydroxy-1,4-dioxo-1,4-dihydronaphthalen-2-yl)-4-methylpent-3-en-1-yl-4-((2-chlorophenethyl)amino)-4-oxobutanoate,* red solid. Yield: 76.6%. m.p. 97.8 °C. ^1^H NMR (600 MHz, CDCl_3_) *δ* = 12.57 (s, 1H), 12.41 (s, 1H), 7.34–7.32 (m, 1H), 7.20–7.14 (m, 5H), 7.01 (s, 1H), 6.01 (dd, *J* = 6.5, 5.2, 1H), 5.62 (s, 1H), 5.10 (t, *J* = 7.3, 1H), 3.51 (dd, *J* = 13.2, 6.8, 2H), 2.92 (t, *J* = 7.0, 2H), 2.79–2.70 (m, 2H), 2.64–2.58 (m, 1H), 2.50–2.41 (m, 3H), 1.68 (s, 3H), 1.56 (s, 3H). ^13 ^C NMR (150 MHz, cdcl_3_) *δ* 177.85, 176.34, 171.84, 170.98, 167.73, 167.20, 147.84, 136.45, 136.17, 134.07, 132.96, 132.73, 131.55, 131.00, 129.59, 128.04, 126.97, 117.54, 111.79, 111.55, 69.81, 39.28, 33.29, 32.82, 30.81, 29.44, 25.76, 17.95. HR-ESI-MS: *m*/*z* [M + H]^+^ calcd for 526.1662.

**19**: *1-(5,8-dihydroxy-1,4-dioxo-1,4-dihydronaphthalen-2-yl)-4-methylpent-3-en-1-yl-4-((4-bromophenethyl)amino)-4-oxobutanoate,* red solid. Yield: 69.4%. m.p. 134.0 °C. ^1^H NMR (600 MHz, CDCl_3_) *δ* = 12.58 (s, 1H), 12.41 (s, 1H), 7.40 (d, *J* = 8.2, 2H), 7.16 (s, 2H), 7.04 (d, *J* = 8.2, 2H), 7.00 (s, 1H), 6.06–5.95 (m, 1H), 5.58 (s, 1H), 5.10 (t, *J* = 7.1, 1H), 3.49–3.43 (m, 2H), 2.77–2.70 (m, 4H), 2.61–2.56 (m, 1H), 2.52–2.47 (m, 1H), 2.42 (t, *J* = 6.7, 2H), 1.67 (s, 3H), 1.56 (s, 3H). ^13 ^C NMR (150 MHz, CDCl_3_) *δ* 177.77, 176.25, 171.85, 170.99, 167.81, 167.28, 147.80, 137.72, 136.18, 132.99, 132.78, 131.65, 131.51, 130.46, 120.35, 117.52, 111.78, 111.54, 69.85, 40.55, 35.05, 33.92, 32.81, 30.79, 29.42, 25.76, 25.58, 17.95. HR-ESI-MS: *m*/*z* [M + H]^+^ calcd for 570.1137.

**20**: *1-(5,8-dihydroxy-1,4-dioxo-1,4-dihydronaphthalen-2-yl)-4-methylpent-3-en-1-yl 4-((4-fluorophenethyl)amino)-4-oxobutanoate,* red solid. Yield: 54.2%. m.p. 116.0 °C. ^1^H NMR (600 MHz, CDCl_3_) *δ* = 12.57 (s, 1H), 12.41 (s, 1H), 7.16 (s, 2H), 7.14–7.10 (m, 2H), 7.00 (s, 1H), 6.97 (t, *J* = 8.6, 2H), 6.05–6.00 (m, 1H), 5.59 (br, 1H), 5.10 (t, *J* = 7.3, 1H), 3.51–3.44 (m, 2H), 2.77–2.70 (m, *J* = 6.8, 4H), 2.66–2.55 (m, 1H), 2.52–2.45 (m, 1H), 2.42 (t, *J* = 6.8, 2H), 1.67 (s, 3H), 1.56 (s, 3H). ^13 ^C NMR (150 MHz, CDCl_3_) *δ* 177.80, 176.28, 171.86, 170.96, 167.78, 167.25, 162.42, 160.79, 147.81, 136.18, 132.98, 132.76, 131.52, 130.13, 130.08, 117.52, 115.45, 115.31, 111.78, 111.54, 69.84, 40.81, 34.82, 32.81, 30.80, 29.44, 25.75, 17.94. HR-ESI-MS: *m*/*z* [M + H]^+^ calcd for 510.1936.

**21**: *1-(5,8-dihydroxy-1,4-dioxo-1,4-dihydronaphthalen-2-yl)-4-methylpent-3-en-1-yl 4-((2-fluorophenethyl)amino)*-*4-oxobutanoate,* red solid. Yield: 75.5%. m.p. 91.0 °C. ^1^H NMR (600 MHz, CDCl_3_) *δ* = 12.57 (s, 1H), 12.41 (s, 1H), 7.21–7.15 (m, 4H), 7.06 (t, *J* = 7.4, 1H), 7.03–6.98 (m, 2H), 6.04–5.98 (m, 1H), 5.64 (br, 1H), 5.10 (t, *J* = 7.2, 1H), 3.49 (q, *J* = 6.7, 2H), 2.83 (t, *J* = 6.9, 2H), 2.75–2.69 (m, 2H), 2.64–2.58 (m, 1H), 2.50–2.46 (m, 1H), 2.43 (t, *J* = 6.9, 2H), 1.67 (s, 3H), 1.56 (s, 3H). ^13 ^C NMR (150 MHz, CDCl_3_) *δ* 177.92, 176.41, 171.83, 170.98, 167.67, 167.14, 162.03, 160.41, 147.86, 136.16, 132.94, 132.71, 131.56, 131.14, 131.11, 128.35, 128.30, 125.74, 125.63, 124.21, 124.19, 117.55, 115.40, 115.26, 111.79, 111.54, 69.80, 39.67, 32.81, 30.79, 29.45, 29.09, 25.75, 17.94. HR-ESI-MS: *m*/*z* [M + H]^+^ calcd for 510.1966.

**22**: *1-(5,8-dihydroxy-1,4-dioxo-1,4-dihydronaphthalen-2-yl)-4-methylpent-3-en-1-yl 4-(diethylamino)-4-oxobutanoate,* red solid. Yield: 78.5%. m.p. 77.0 °C.^1^H NMR (600 MHz, CDCl_3_) *δ* = 12.57 (s, 1H), 12.43 (s, 1H), 7.19–7.14 (m, 2H), 7.05 (s, 1H), 6.02–5.99 (m, 1H), 5.12 (t, *J* = 7.2, 1H), 3.36 (q, *J* = 7.1, 1H), 3.31 (q, *J* = 7.1, 2H), 2.75 (t, *J* = 6.7, 2H), 2.65–2.58 (m, 3H), 2.52–2.47 (m, 1H), 1.67 (s, 3H), 1.56 (s, 3H), 1.18 (t, *J* = 7.1, 3H), 1.08 (t, *J* = 7.1, 3H). ^13 ^C NMR (150 MHz, CDCl_3_) *δ* 178.85, 177.39, 172.17, 169.81, 166.79, 166.27, 148.18, 135.95, 132.60, 132.37, 131.92, 117.70, 111.82, 111.57, 69.65, 41.73, 40.28, 32.78, 29.43, 27.85, 25.75, 17.94, 14.12, 13.04. HR-ESI-MS: *m*/*z* [M + H]^+^ calcd for 444.2024.

**23**: *1-(5,8-dihydroxy-1,4-dioxo-1,4-dihydronaphthalen-2-yl)-4-methylpent-3-en-1-yl 4-(dihexylamino)-4-oxobutanoate1-(5,8-dihydroxy-1,4-dioxo-1,4-dihydronaphthalen-2-yl)-4-methylpent-3-en-1-yl 4-(dihexylamino)-4-oxobutanoate*, red solid. Yield: 53.8%. m.p. 78.3 °C. ^1^H NMR (600 MHz, CDCl_3_) *δ* = 12.56 (s, 1H), 12.44 (s, 1H), 7.19–7.14 (m, 2H), 7.05 (s, 1H), 6.00 (dd, *J* = 6.3, 5.0, 1H), 5.12 (t, *J* = 7.2, 1H), 3.29–3.25 (m, 2H), 3.22–3.18 (m, 2H), 2.74 (t, *J* = 6.7, 2H), 2.65–2.58 (m, 3H), 2.51–2.45 (m, 1H), 1.67 (s, 3H), 1.55 (s, 3H), 1.32–1.21 (m, 16H), 0.90–0.83 (m, 6H). ^13 ^C NMR (150 MHz, CDCl_3_) *δ* 178.92, 177.47, 172.18, 170.11, 166.71, 166.19, 148.19, 135.94, 132.58, 132.34, 131.96, 117.71, 111.81, 111.56, 69.62, 47.82, 46.17, 32.78, 31.60, 31.50, 29.69, 29.48, 28.87, 27.90, 27.71, 26.70, 26.60, 25.77, 22.59, 17.95, 14.04, 13.99. HR-ESI-MS: *m*/*z* [M + H]^+^ calcd for 556.3284.

**24**: *1-(5,8-dihydroxy-1,4-dioxo-1,4-dihydronaphthalen-2-yl)-4-methylpent-3-en-1-yl (Z)-4-morpholino-4-oxobut-2-enoate,* red solid. Yield: 49.6%. m.p. 104.0 °C. ^1^H NMR (600 MHz, CDCl_3_) *δ* = 12.57 (s, 1H), 12.40 (s, 1H), 7.17 (s, 2H), 7.02 (s, 1H), 6.61 (d, *J* = 11.9, 1H), 6.12 (d, *J* = 11.9, 1H), 6.09 (dd, *J* = 6.3, 4.9, 1H), 5.10 (t, *J* = 7.1, 1H), 3.74–3.58 (m, 4H), 2.91 (dd, *J* = 16.5, 4.5, 2H), 2.76 (dd, *J* = 16.6, 4.6, 2H), 2.67–2.61 (m, 1H), 2.53–2.45 (m, 1H), 1.67 (s, 3H), 1.55 (s, 3H). ^13 ^C NMR (150 MHz, CDCl_3_) *δ* = 180.62, 179.13, 174.00, 171.50, 169.02, 168.50, 150.01, 138.03, 137.29, 134.71, 134.49, 133.85, 124.11, 119.65, 113.80, 113.56, 71.77, 68.83, 68.46, 47.61, 44.06, 29.71, 27.78, 19.96. HR-ESI-MS: *m*/*z* [M + H]^+^ calcd for 456.1628.

### 2.2. Cell culture

MGC-803, SGC-7901, U87 and SMMC-7721 cell lines were cultured in DMEM supplemented with 10% foetal bovine serum, 100 IU/mL penicillin/streptomycin. The cells were incubated in an atmosphere containing 5% CO_2_ at 37 °C.

### 2.3. Cell viability assay

MGC-803, SGC-7901, U87 and SMMC-7721 cells were plated in 96-well plate at 7000 per well. Cells were incubated with the different concentrations of the compounds for 48 h. Subsequently, MTT (0.5 mg/mL) were used to test the cell viability, then 150 μL DMSO was added to each well after cells were incubated for 4 h and the absorbance was measured at 492 nm.

### 2.4. Apoptosis assay

After the indicated treatments for 48 h, cells were collected, washed three times with cold PBS, centrifuged and resuspended with 400 μL AnnexinV binding buffer per tube. Then the cells were stained with 5μLAnnexinV-FITC and incubated in the dark for 10 min on ice. Finally, 10 μL PI staining solution was added, and cells were detected by the flow cytometry after incubated in the dark for 5 min. Flowjo 7.6.1 software was used to analyse the cell apoptosis.

### 2.5. Colony formation assay

SGC-7901 cells (8000/well) were seeded in 6-well plate, and treated with indicated concentrations of compound **12** (2 μM, 4 μM, 8 μM) for 12 d. The medium was removed and methanol (500 μL/well) was added to fix at the cells for 3 min. Then cells were stained with giemsa working fluid for 15 min and washed with PBS. The number of colonies was counted.

### 2.6. Acridineorange (AO) staining

PH-sensitive Acridine orange (AO) was used to label acidic vesicles. Human gastric cancer cells were seeded in laser confocal dishes at 10,000/ml. After indicated treatment of compound **12** (1 μM, 2 μM, 4 μM) for 48 h, cells were stained with AO (1 μg/ml) for 15 min at the cell culture incubator. After the cells were washed by PBS, pictures were acquired under laser confocal microscope.

### 2.7. GFP-LC3 transfection

A plasmid GFP-tagged LC3 reporter gene (0.8 μg/500 μL) was transfected into SGC-7901 cells by Lipofectamine 2000, then cells were incubated for 24 h. After different treatments for 24 h, cells were fixed with 4% paraformaldehyde for 20 min, and washed three times with PBS. Finally, Laser confocal dishes were analysed using confocal microscope.

### 2.8. Western blot analysis

RIPA lysis buffer was used to dissociate the treated cells. Cells were lysed and the supernatant was harvested after centrifuged for 30 min. The cell proteins were separated using Sodium dodecyl sulphate-polyacrylamide gel electrophoresis, and then they were transferred to the PVDF membranes. The membranes were blocked with skimmed milk solution (5%) for 2 h, then incubated with primary antibodies (1:1000, cst) for 20 h at 4 °C. The membranes were detected by chemiluminescence reagent after probed with the secondary antibody for 1 h.

### 2.9. Statistical analysis

Data were represented as the mean ± SD (standard deviation). GraphPad Prism 5.0 software (GraphPad Software, La Jolla, CA) were used to analyse significance by one-way ANOVA. *p* < 0.05 was expressed a significant difference.

## 3. Results

### 3.1. Scaffold design

**Figure 2.**
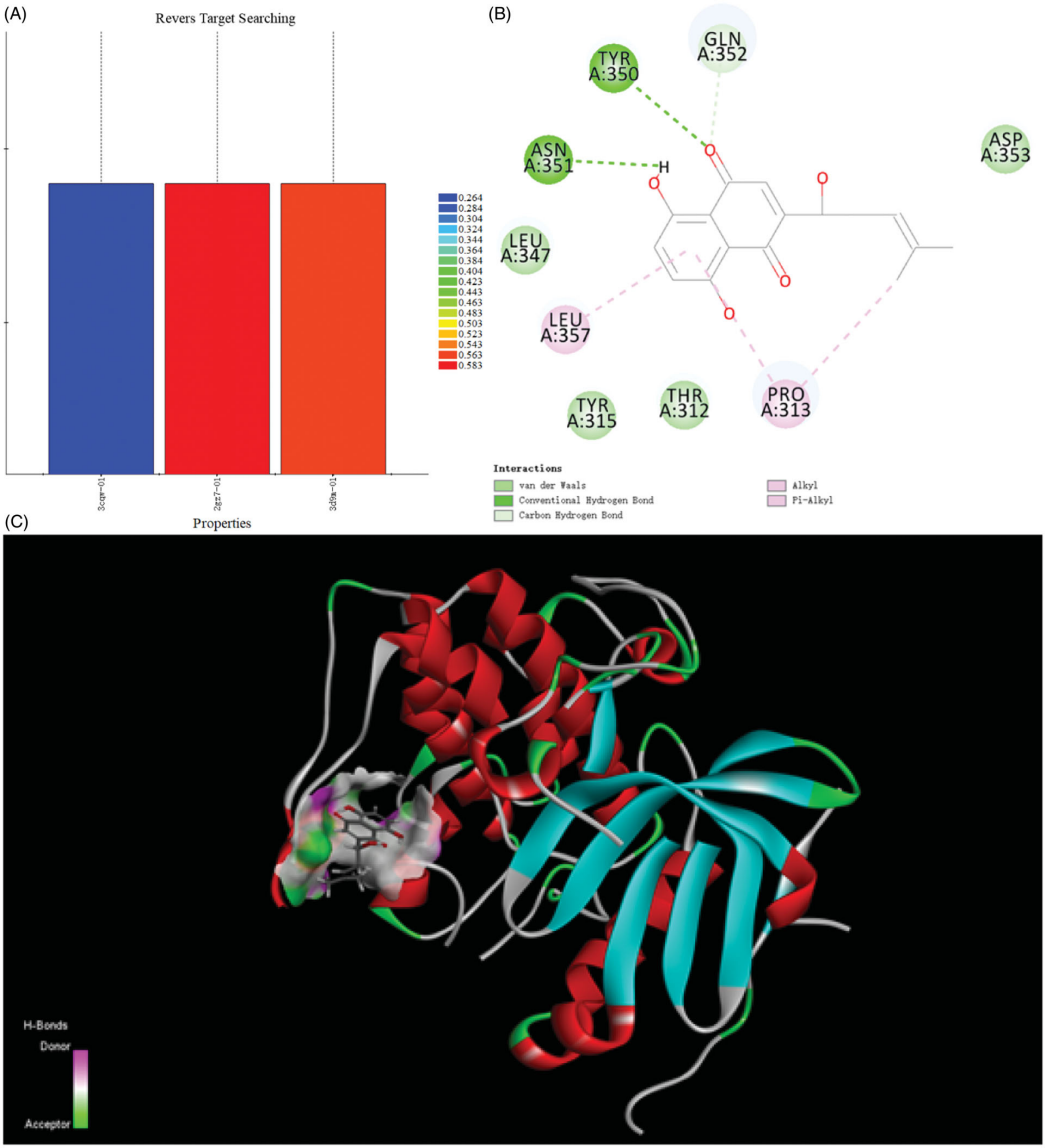
Ligand profiler and docking result of naphthoquinone moiety.

Discovery Studio 2018 was used as computation programme and naphthoquinone moiety as ligand to execute reverse targets searching programme, targets were searched to suit the pharmacophores of protein active sites in the database. Comparison with protein data bank, the results indicated the PI3K/Akt signal pathway was the target of naphthoquinone moiety. The lower most CDDOCKER energy of all conformations is 6.13917 kcal/m. The moiety interacted with protein active site through those forces, containing van der Waals force, conventional hydrogen bond, carbon hydrogen bond, alkyl and Pi-alkyl interactions. Van der Waals force formed between naphthoquinone and the receptors (Thr312, Tyr315, Leu347, Gln352). There was a hydrogen bond interaction between Asn351 and compound. Alkyl and Pi-Alkyl interacts with the aryl ring and the alkyl group via Leu357 and Pro313 (Figure 2).

**Figure 3.**
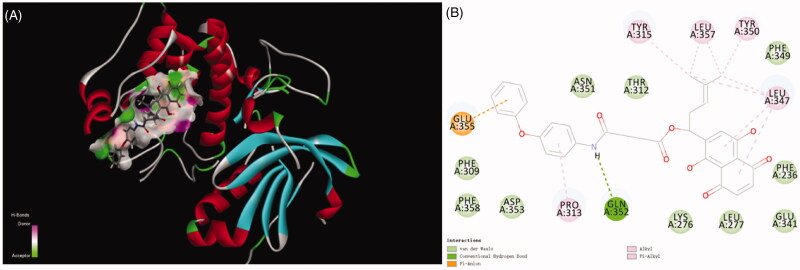
Docking result of compound **12** with Akt.

Title compound **12** has five kinds of interactions, such as hydrogen bonds, Pi-Anion force, Pi-alkyl interactions, and conventional van der Waals force which were formed with Phe309, Phe349, Phe358, Asp353, Asn351, Thr312, Lys276, Leu277, Glu341 and Phe236. Between the nitrogen of the compound and Gln352 formed the hydrogen bond. There also existed a Pi-anion interaction between aryl ring and the Glu355. Alky and Pi-Alkyl interactions were formed between aryl rings and Pro313, Tyr315, Leu357, Tyr350 and Leu347. Compared with naphthoquinone moiety, there was more stronger interaction of compound **12** than that of it. The differences of CDDOCKER energy also proved this. (The lower most CDDOCKER Energy of compound **12** is −15.7597 kcal/m and naphthoquinone is 6.13917 kcal/m). These data indicated that the combination of title compound **12** with the cavity site of Akt (PDB ID:3CQW) is spontaneous, which was more easier than that of naphthoquinone moiety (Figure 3).

### 3.2. Chemistry

**Scheme 1.**
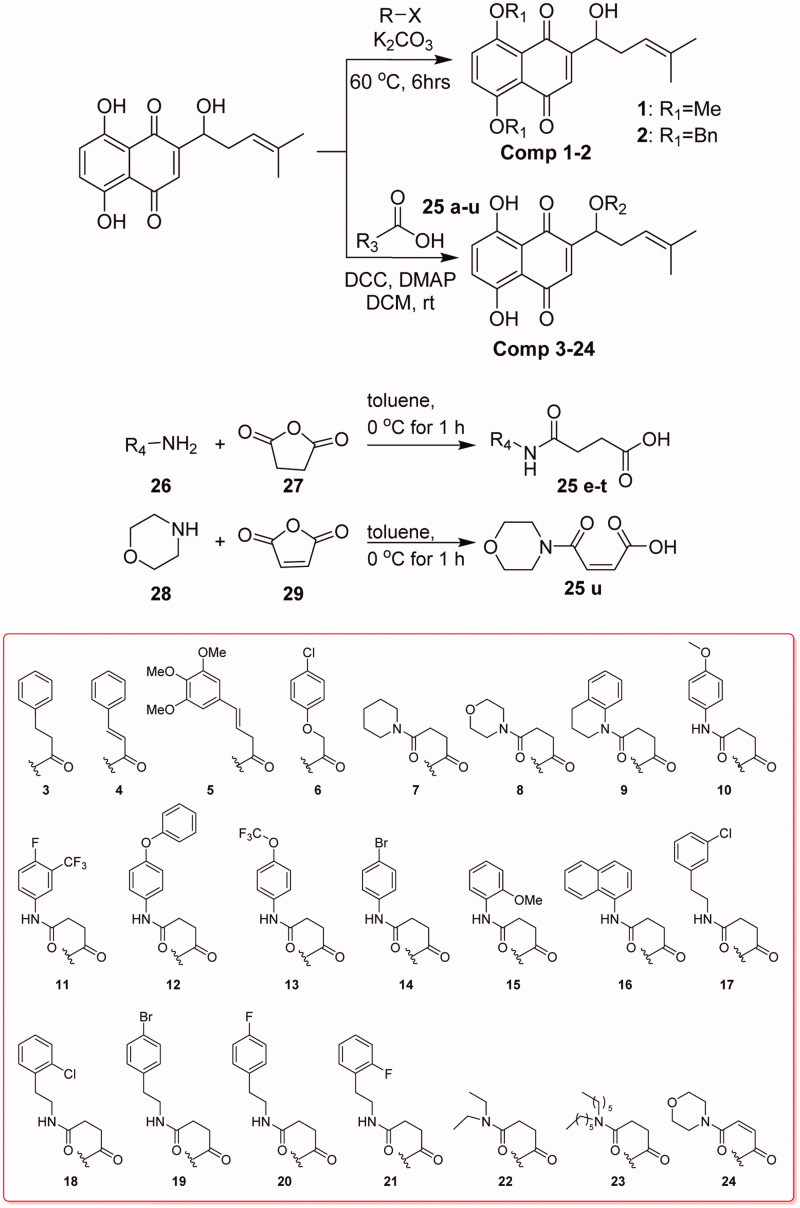
Synthesis of title compounds **1**–**24.**

Compounds **1** and **2** can be obtained by substitution of halides with phenol hydroxyls of naphthoquinone. The condensation reaction between hydroxyl group on side-chain of naphthoquinone and the related acids can get compounds **3**–**24**. Compounds **25e**–**u** were obtained from succinic anhydride or maleic anhydride after ring-opening reaction (Scheme 1).

### 3.3. Evaluation of anti-proliferation activity

**Figure 4.**
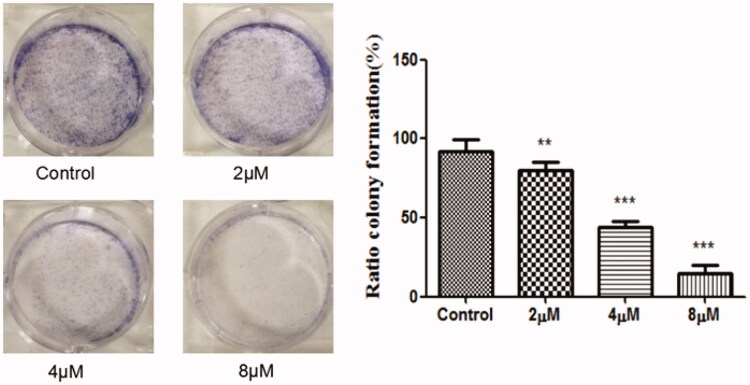
The SGC-7901 cells formed colonies (counted with Image-ProPlus) after treated with various concentrations of the compound **12** for 12 d. **p < 0.01, ***p < 0.001 compared with the control group.

Compounds **1–24** were detected for anticancer activities against SGC-7901, MGC-803, SMMC-7721, U-87 cell lines in *vitro* by the MTT assay, and ADM was used as the positive control^34^. From Table 1, when the phenolic hydroxyls of naphthoquinone moiety were converted to ether, the activity was missing. Compounds **9, 11, 12**, **13**, **17** and **20** have good activity which showed that hydroxyl group on side-chain of naphthoquinone moiety was modifiable and the introducing of hydrogen-bond donor, acceptor and hydrophobic group could benefit the activity. Compound **12** displayed high activity against the MGC-803, SGC-7901 and U87 cells with the IC_50s_ of 4.07, 4.09 and 3.85 μM, respectively. So colony formation assay of this compound (Figure 4) was used to investigate the effect of it on cell proliferation in SGC-7901 cells. The experiment examined the ability for producing colonies after the cells were treated with the cell death agents^35^^,^^36^. The results exhibited that the colonies were decreased with the increased concentration of the title compound compared with the control group, which revealed this compound could inhibit the cell proliferation.

**Table 1.** *In vitro* anticancer activities of compounds **1**–**24** against U 87, SMMC-7721, SGC-7901, MGC-803 cell lines.

Compounds	IC_50_ μM (*n* = 3)				
	U87	SMMC-7721	SGC-7901	MGC-803	L02
**1**	–	–	–	–	–^a^
**2**	–	–	–	–	–
**3**	8.0 ± 0.85	15.6 ± 3.3	10.6 ± 4.3	4.5 ± 1.1	13.6 ± 1.5
**4**	65.7 ± 2.7		75.1 ± 0.4	–	–
**5**	–	–	–	–	–
**6**	–	–	–	–	–
**7**	9.68 ± 2.3	14.4 ± 1.5	33.6 ± 2.1	9.7 ± 2.3	36.8 ± 4.1
**8**	10.0 ± 1.6	16.7 ± 1.8	20 ± 3.9	10.4 ± 0.7	>50
**9**	4.45 ± 0.66	12.3 ± 0.1	8.6 ± 2.5	3.1 ± 0.1	12.1 ± 0.8
**10**	12.3 ± 2.8	12.4 ± 2.6	11.1 ± 1.2	7.9 ± 1.8	11.9 ± 0.6
**11**	3.75 ± 0.77	9.5 ± 0.8	2.25 ± 1.5	8.4 ± 3.1	4.0 ± 0.1
**12**	3.8 ± 0.2	20.6 ± 0.4	4.1 ± 2.6	4.1 ± 0.6	>50
**13**	3.46 ± 0.31	16.9 ± 2.2	7.8 ± 3.7	10.2 ± 2.9	15.8 ± 1.1
**14**	–	14.8 ± 0.1	10.7 ± 1.8	10.2 ± 1.3	16.2 ± 1.7
**15**	–	–	–	–	–
**16**	5.41 ± 0.67	24.3 ± 1.7	5.6 ± 2.6	5.9 ± 2.6	13.0 ± 2.4
**17**	3.8 ± 1.7	15.9 ± 0.2	20.8 ± 0.5	6.7 ± 2.4	11.4 ± 1.8
**18**	7.7 ± 4.4	17.9 ± 1.6	23.7 ± 0.5	6.6 ± 4.7	20.2 ± 2.0
**19**	9.1 ± 2.5	17.9 ± 1.0	7.6 ± 2.3	5.7 ± 2.4	8.5 ± 0.4
**20**	5.2 ± 1.4	18.6 ± 3.6	13.3 ± 0.6	3.5 ± 1.4	12.3 ± 0.5
**21**	6.1 ± 0.6	15.4 ± 0.4	14.8 ± 1.6	6.9 ± 3.9	16.0 ± 2.2
**22**	40.7 ± 4.7	46.5 ± 1.7	19.8 ± 1.8	9.4 ± 0.4	>50
**23**	–	–	–	–	–
**24**	20.6 ± 0.6	50.1 ± 0.8	38.5 ± 0.7	40.8 ± 1.9	–
ADM	0.57 ± 0.32	0.46 ± 0.63	0.72 ± 0.12	0.48 ± 0.06	–

^a^Inactive at 100 µM (highest concentration tested).

### 3.4. Metabolic stability assay in human liver microsomes

To initially evaluate the stability of compound **12**, we then tested the liver microsome stability of this compound. The Mean % Parent Remaining of compound **12** in metabolic stability test were 100, 95, 85, 80, and 70 at 0, 15, 30, 45, and 60 min, respectively. The calculated clearance rate was >110 μL/min/mg and the half-life (*t*_1/2_) was more than 60 min which indicated the acceptable stability.

### 3.5. Induces apoptosis

**Figure 5.**
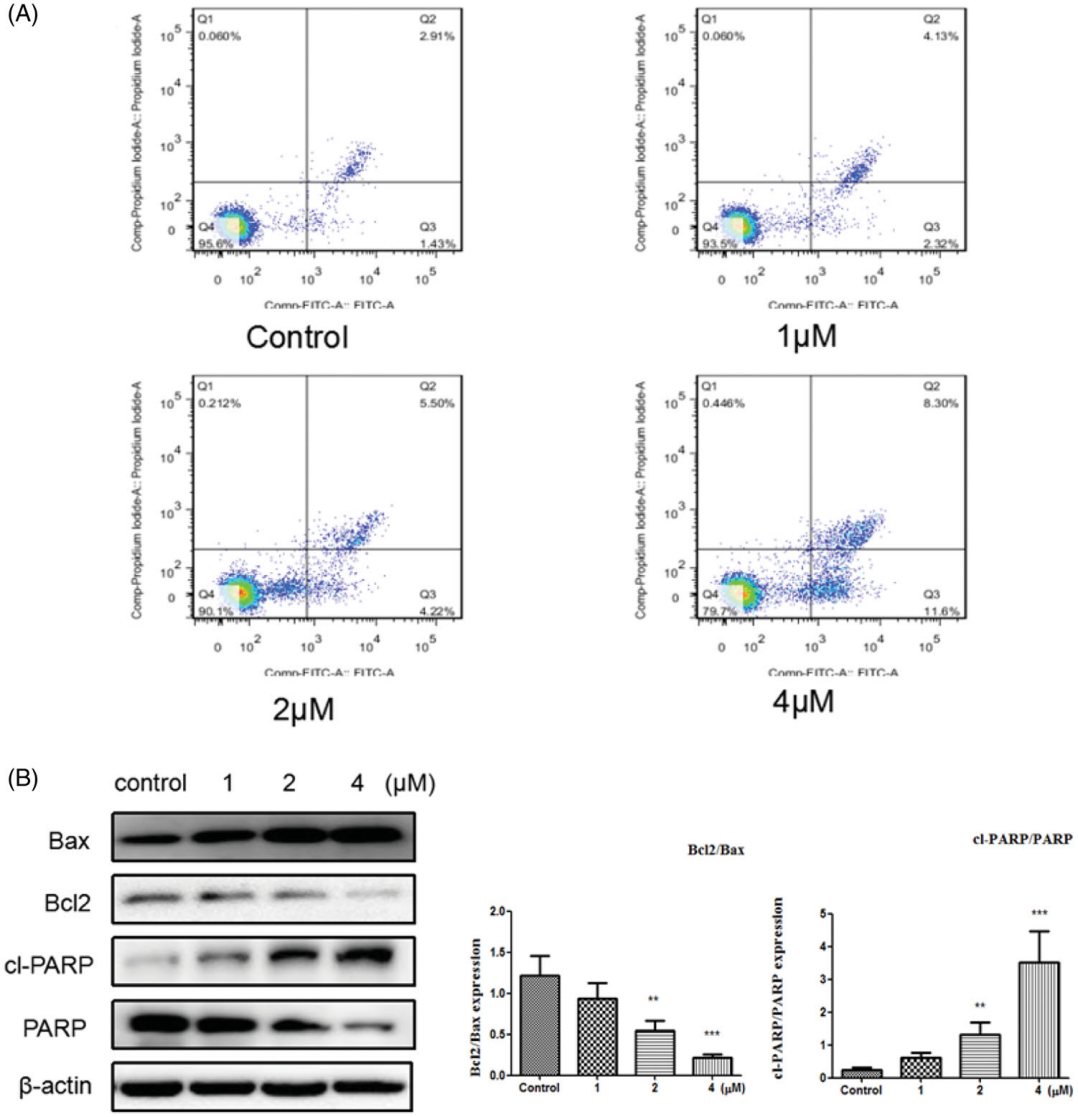
(A) Apoptosis ratio was detected by Annexin V/PI staining on the SGC-7901 cells treated with various concentration of title compound **12** (1, 2, 4 μM). (B) Western blots were used to detect the Bax, Bcl-2, cleaved PARP and full length PARP protein expression. ***p < 0.001, **p < 0.01 compared with the control group.

Apoptosis is one of the main methods that result in cell death^37–39^. In this study, Annexin V-FITC/PI kit was used to evaluate the cell apoptosis. From Figure 5(A), it can be see that after the cells were treated with the compound **12** (1, 2, and 4 μM) for 48 h, the total apoptosis was increased to 6.45%, 9.72%, and 19.9%, respectively, compared to the control group (4.34%). These results indicated that compound **12** induced apoptosis was associated with a dose dependent. To further, investigate whether the cell apoptosis induced by title compound, Western blot analysis was used to measure the effect of the compound on apoptosis of SGC-7901 cell. As reported that apoptosis pathway could be regulated by the activation of PARP and Bcl2 family proteins^40^^,^^41^. It can be found that the levels of Bax and cleaved PARP were up-regulated and the level of Bcl2 was down-regulated by the increased concentration of compound **12**, which suggested that compound could promote apoptosis in SGC-7901l cells (Figure 5(B)).

### 3.6. Induces autophagy

**Figure 6.**
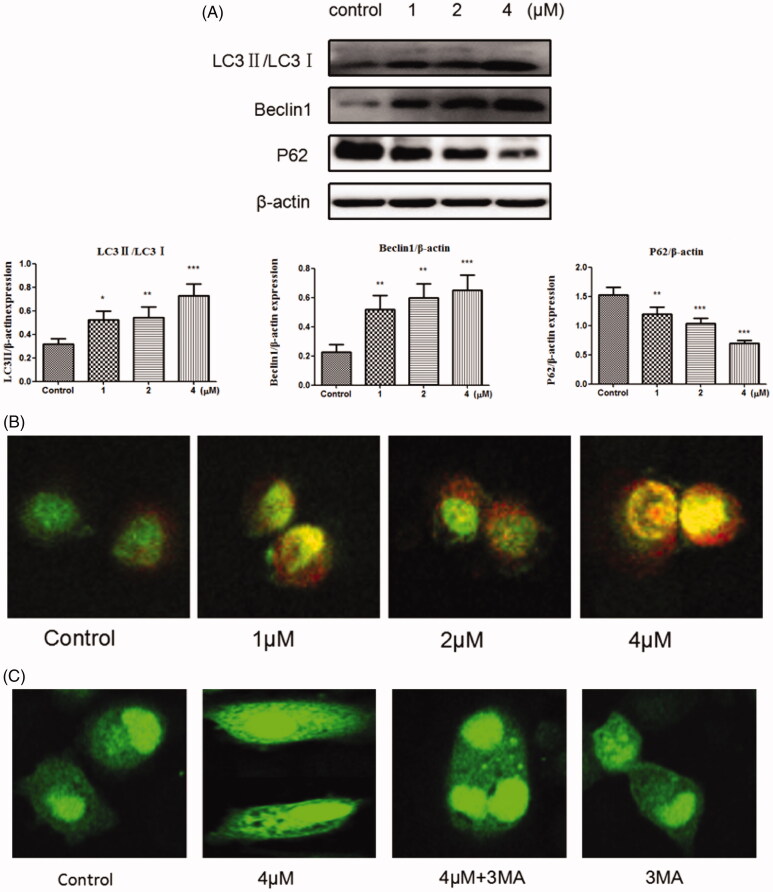
(A) Western blots assay examined the expressions of LC3-II/LC3-, P62, Beclin1. *p < 0.05, **p < 0.01, ***p < 0.001 compared with the control group. (B) SGC-7901 cells were stained with AO after exposed to compound **12** for 48 h, then detected by the confocal microscopy at 200×. (C) SGC-7901 cells were transfected with GFP-LC3 plasmid, and treated with compound **12** alone (4 μM), 3MA (500 μM, pro-incubated for 1 h), compound **12** and 3MA, then observed under a confocal microscopy at 200×.

Autophagy is a process of lysosomal degradation that can deliver the cytoplasmic cargo to the lysosome^42^. To assess whether compound **12** could promote autophagy on SGC-7901 cells, the expressions of autophagy-related proteins were examined. Autophagy will be switched on when LC3-I was converted into LC3-II, and the level of LC3-II involves in the formation of autophagosome. Meanwhile p62 is a marker of the degradative lysosome^43^^,^^44^. As shown in Figure 6(A), compound can decrease the level of P62 and increase the expressions of LC3II and Beclin1. The preliminary results suggest that title compound may promote autophagy.

Another typical feature of autophagy is the accumulation of the acidic autophagic vacuoles (AVOs)^45^^,^^46^. To verify the development of AVOs, Acridine Orange (AO) staining was used to examine whether the acidic vesicular organelles in SGC-7901 cells were increased. AO produces red fluorescence when it accumulates in acidic regions such as autophagy lysosomes and lysosomes and produces bright green fluorescence in the cytoplasm and the nucleus^47^. As it can be seen in Figure 6(B), red fluorescently labelled vesicle acidic accumulated obviously as the increased concentrations of title compound (1, 2, and 4 μM). GFP-LC3 indicating technique was also used to detect the autophagy at the same time. When autophagy is formed, multiple bright green fluorescent spots will form as the GFP-LC3 fusion protein can translate to autophagosome membrane^48^. From Figure 6(C), compared to the blank control group, GFP-LC3 puncta were increased after cells treated with compound **12** (4 μM) meanwhile decreased after treated with compound **12** (4 μM) and 3-MA (500 μM). This further proved that title compound can promote autophagy.

### 3.7. Role of autophagy regulation

**Figure 7.**
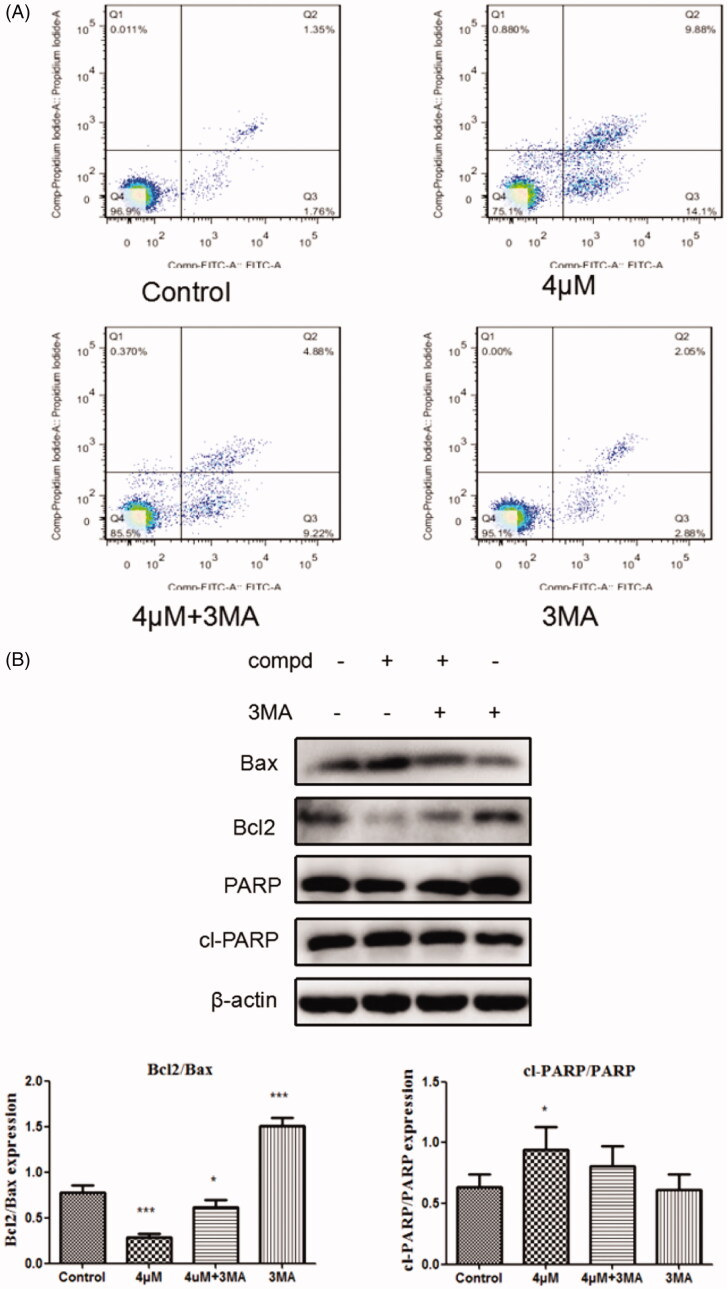
(A) Cells were treated with compound **12** alone (4 μM), 3MA (500 μM, pro-incubated for 1 h), compound **12** combined with 3MA and the Annexinv-FITC staining was used to evaluate the apoptosis ratio. (B) The activation of Bax, Bcl2, cl-PARP were determined by the western blot after SGC-7901 cells were exposed to compound **12** for 48 h with or without of 3MA (500 μM). *p < 0.05, ***p < 0.001 compared with the control group.

The connection between apoptosis and autophagyis complicated because autophagy can promote apoptosis^49^^,^^50^, and can also suppress apoptosis^51^^,^^52^. To investigate whether autophagy has an impact on cell apoptosis induced by title compound, the cells were treated with compound **12** (4 μM) for 48 h co-treatment with or absence of the 3-MA (500 μM) in the apoptosis assays (Figure 7(A)) detected by the flow cytometry. Compared with compound **12**, the apoptosis rate of treatment group cells in the presence of 3-MA decreased. At the same time, the levels of apoptosis-related proteins were examined in Western blot assay (Figure 7(B)), it was found that the up-regulation of apoptosis protein Bax and cl-PARP and the down-regulation of Bcl-2 was inhibited in the group with 3-MA. These results indicated that cell autophagy induced by compound **12** might promote the apoptosis of SGC-7901 cells.

### 3.8. Suppression of activation of the PI3K/AKT/MTOR pathway

**Figure 8.**
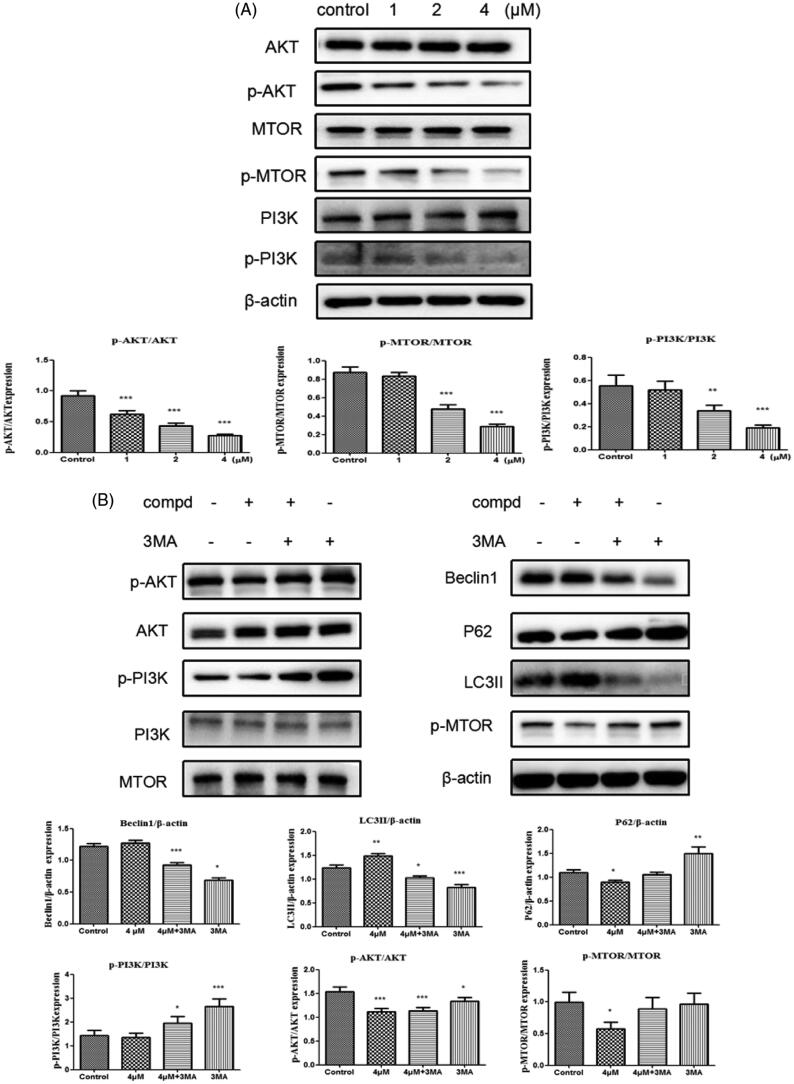
(A) Western blots were performed to observe the PI3K, p-PI3K, AKT, p-AKT, mTOR, p-MTOR protein expression incubated with compound **12** (1 μM, 2 μM, 4 μM) for 48 h. *p < 0.05, **p < 0.01, ***p < 0.001 compared with the control group. (B) The expression levels of LC3-II, Beclin-1, P62 and PI3K signalling were analysed by western blotting assay with or without pre-treatment of 3MA (500 μM). *p < 0.05, ***p < 0.001 compared with the control group.

As a therapeutic target for cancer, the PI3K/AKT/MTOR signalling pathway participates in regulating autophagy^53–56^. Consequently, Western blot experiment was used to explore the impact of compound **12** on the level of phospho-PI3K, phospho-AKT and phospho-mTOR. After cells were treated with title compound for 48 h, the expression of p-PI3K, p-AKT and p-MTOR were decreased in a dose-dependent manner (Figure 8(A)). 3-MA (3-methyladenine) is also a PI3K inhibitor^57^, so compound **12** (4 μM) with 3-MA (500 μM) were used to treated the cells. The experimental data showed that the ratio of p-PI3K/PI3K, p-AKT/AKT and p-MTOR/MTOR increased in the experiment of compound **12** with 3-MA compared with compound **12** alone (Figure 8(B)). These results revealed title compound could induce cell autophagy via inhibiting PI3K pathway.

## 4. Conclusion

According to result of ligand profiler and docking, a series of naphthoquinone derivatives were designed and synthesised. The preliminary activity results showed that several compounds had good anticancer activity. The anticancer mechanism of one compound against SGC-7901 cells was investigated further. The expression of LC3-II and Beclin1increased and the expression of P62 decreased after treated with this compound, which means that this compound helps promote the cell autophagy. Moreover, in Western blot and GFP-LC3 studies, the level of LC3-II/LC3-I decreased and autophagosome puncta was reduced after pre-treatment with 3-MA, which further verify our view. In addition, the cell apoptosis induced by this compound was inhibited and the cell viability was increased when the cell autophagy was blocked by 3-MA. Sequentially, the levels of p-MTOR, p-AKT, and p-PI3K were suppressed after incubated with compound, and these indicated that title compound could negatively regulate the PI3K pathways. In conclusion, title compound could inhibit cancer cell growth by promoting autophagy.

## Supplementary Material

Supplemental Material
