# Supplementary material for: Design, synthesis and anticancer activity of naphthoquinone derivatives
Source: J Enzyme Inhib Med Chem. 2020 Mar 23;35(1):773–85. doi: 10.1080/14756366.2020.1740693 (PMC7144209; doi:10.1080/14756366.2020.1740693)
Supplement: Supplemental Material [file IENZ_A_1740693_SM6950.pdf]

Supporting Information

Design, synthesis and anticancer activity of naphthoquinone derivatives

Xiao-bao Shen[a,b], Yang Wang[a], Xuan-zhen Han[b], Liang-quan Sheng[b], Fu-fangWu[a,b,*], Xin-hua Liu[a,*]

Contents

^{1}H NMR and ^{13}C NMR…..S2-S70

^{1}H NMR and ^{13}C NMR

2-(1-hydroxy-4-methylpent-3-en-1-yl)-5,8-dimethoxynaphthalene-1,4-dione (1, known compound)

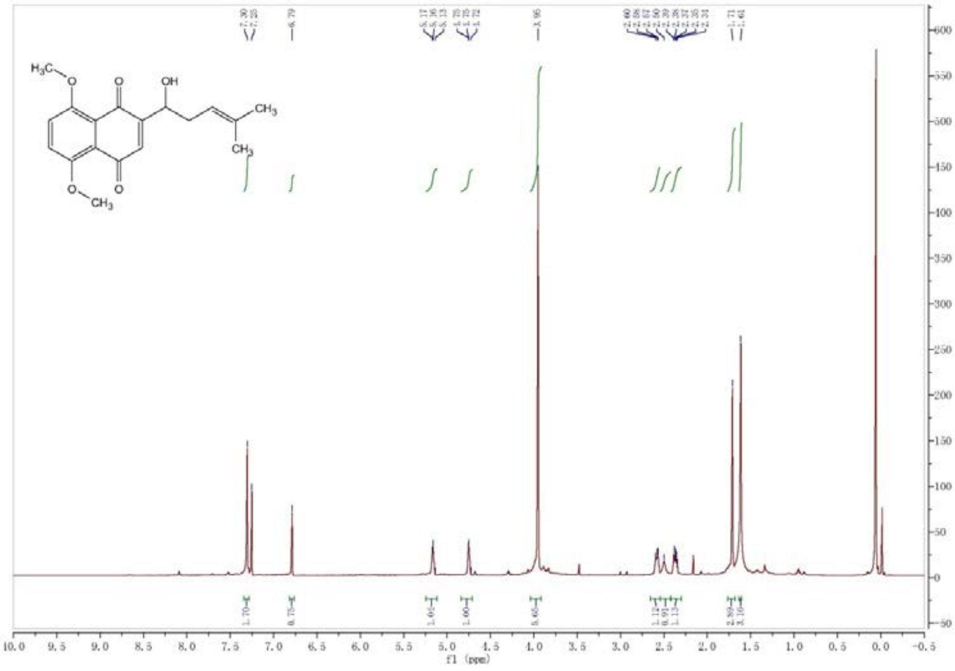

^{1}H NMR spectrum

5,8-bis(benzyloxy)-2-(1-hydroxy-4-methylpent-3-en-1-yl)naphthalene-1,4-dione (2)

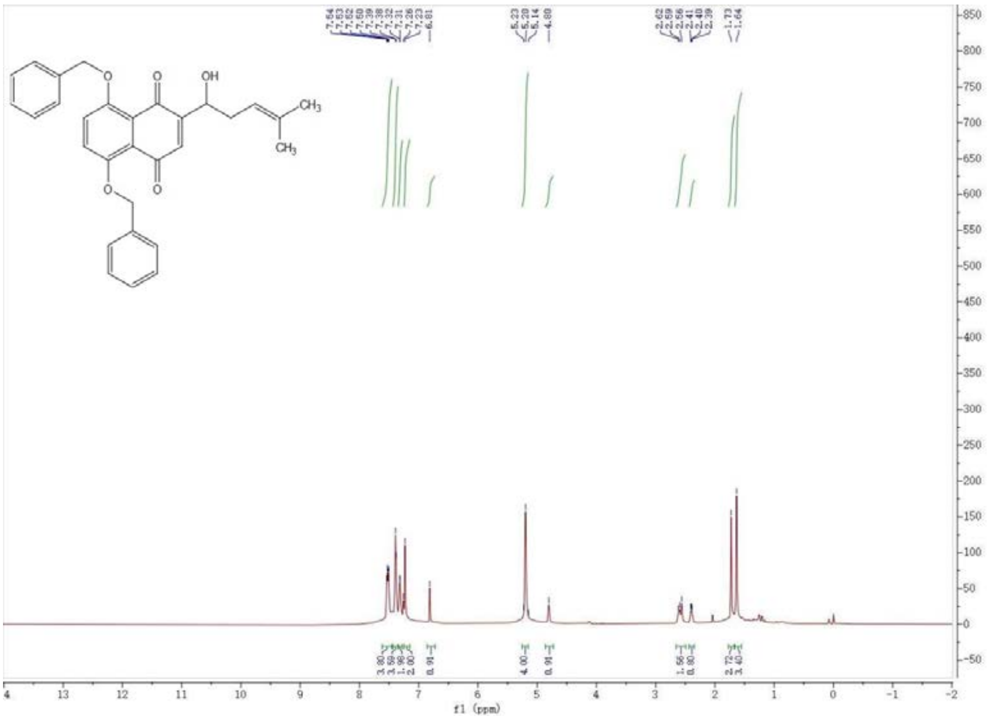

^{1}H NMR spectrum

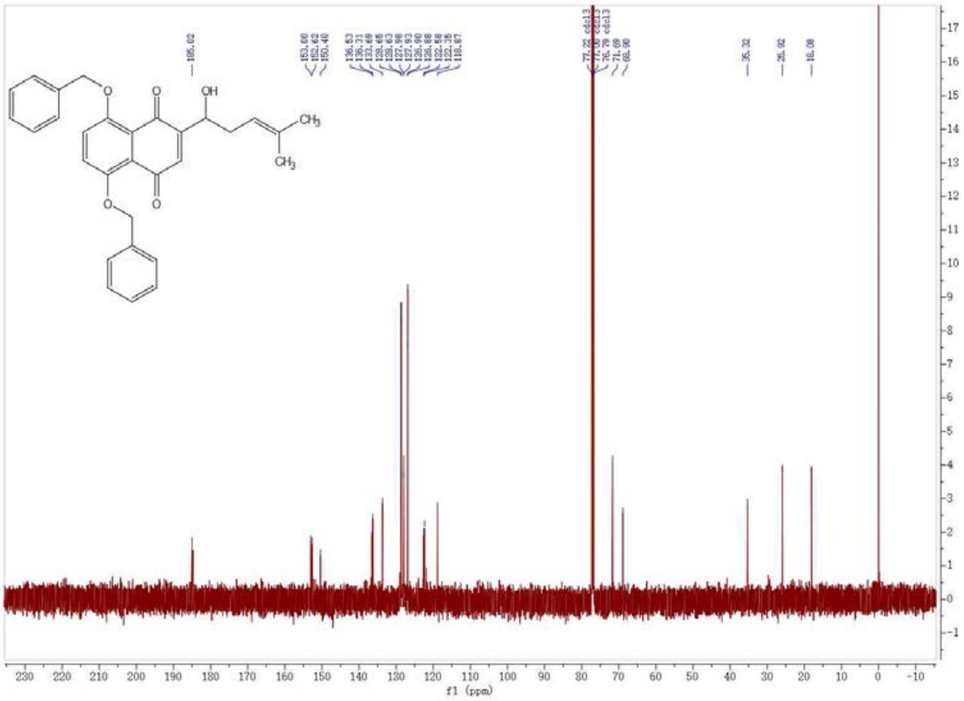

^{13}C NMR spectrum

1-(5,8-dihydroxy-1,4-dioxo-1,4-dihydronaphthalen-2-yl)-4-methylpent-3-en-1-yl-3-phenylpropanoate (3)

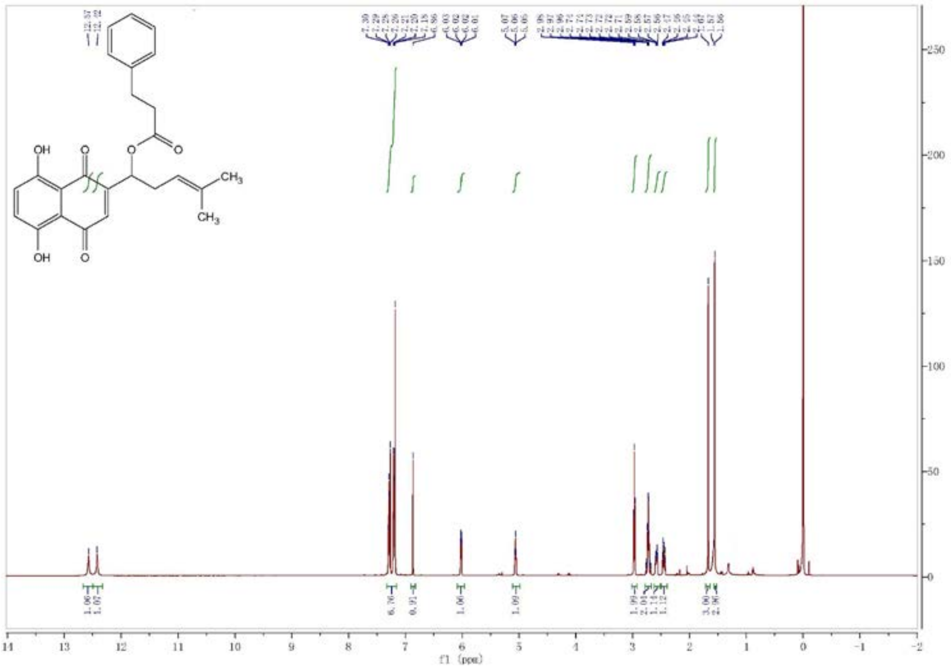

^{1}H NMR spectrum

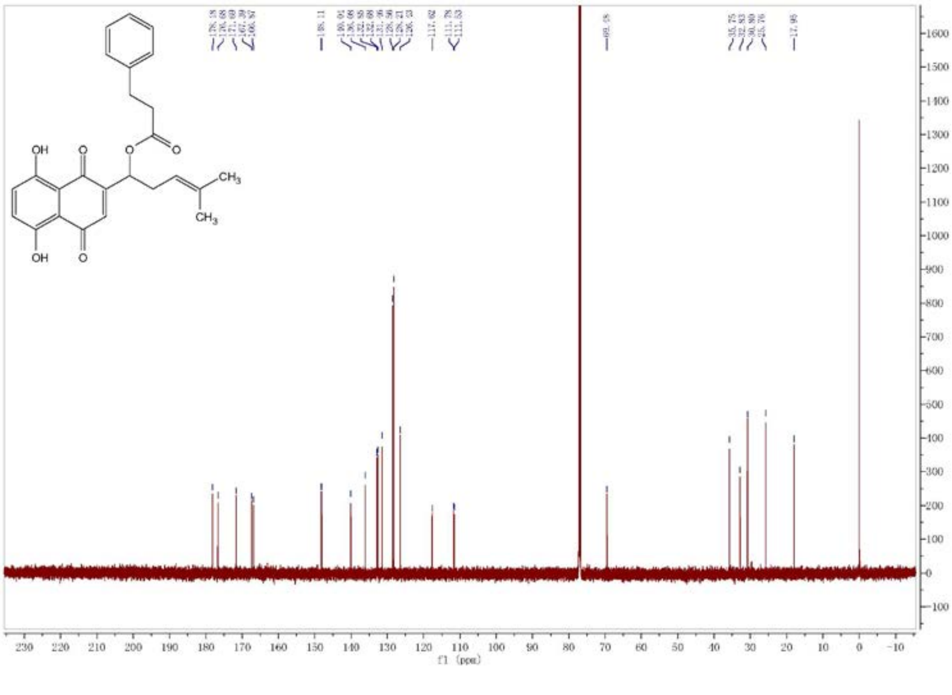

^{13}C NMR spectrum

1-(5,8-dihydroxy-1,4-dioxo-1,4-dihydronaphthalen-2-yl)-4-methylpent-3-en-1-yl cinnamate (4)

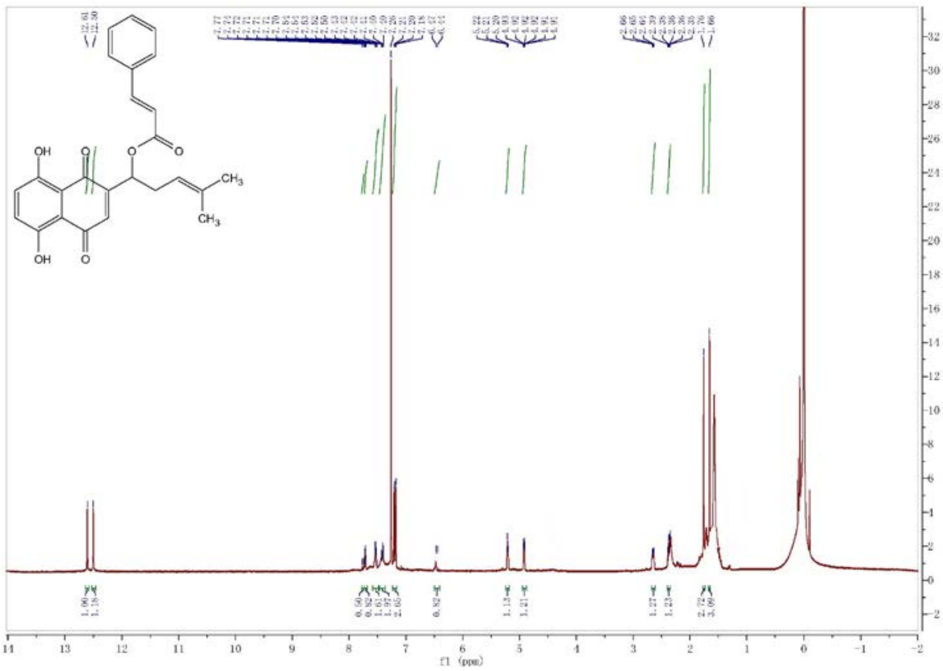

^{1}H NMR spectrum

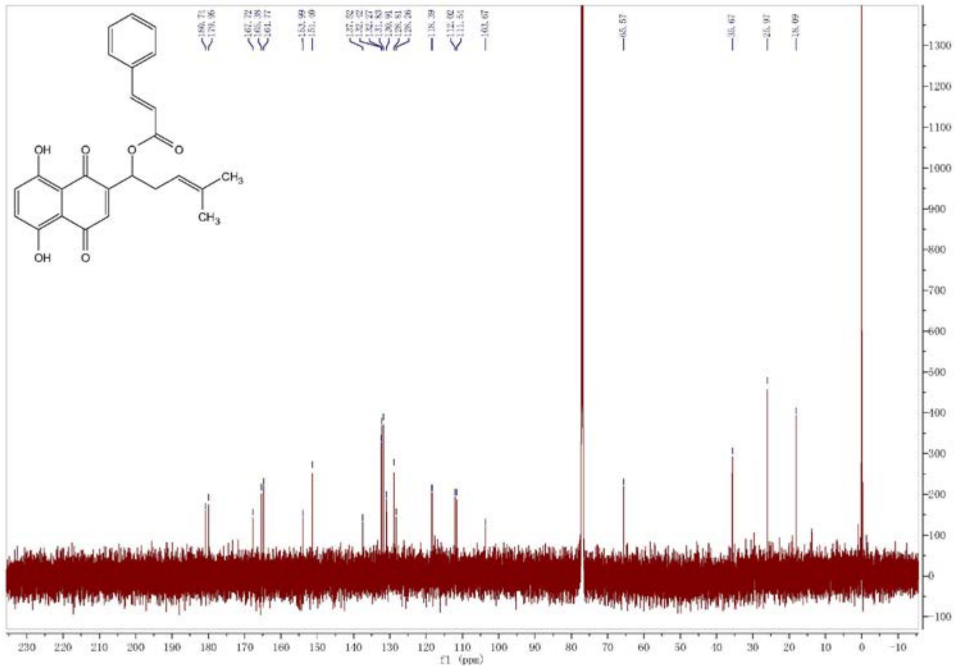

^{13}C NMR spectrum

1-(5,8-dihydroxy-1,4-dioxo-1,4-dihydronaphthalen-2-yl)-4-methylpent-3-en-1-yl (E)-4-(3,4,5-trimethoxyphenyl)but-3-enoate (5)

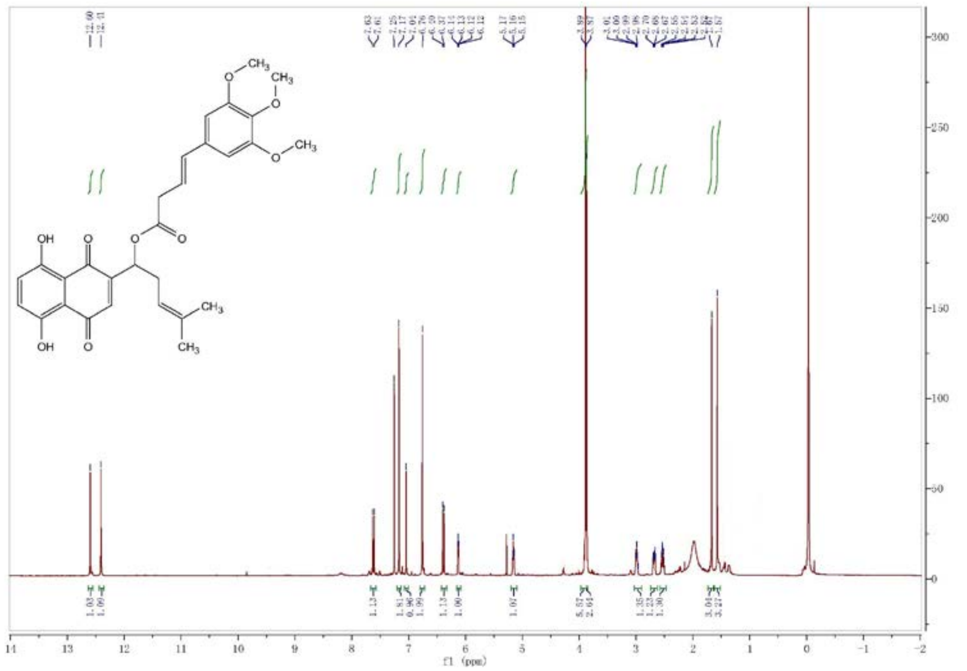

^{1}H NMR spectrum

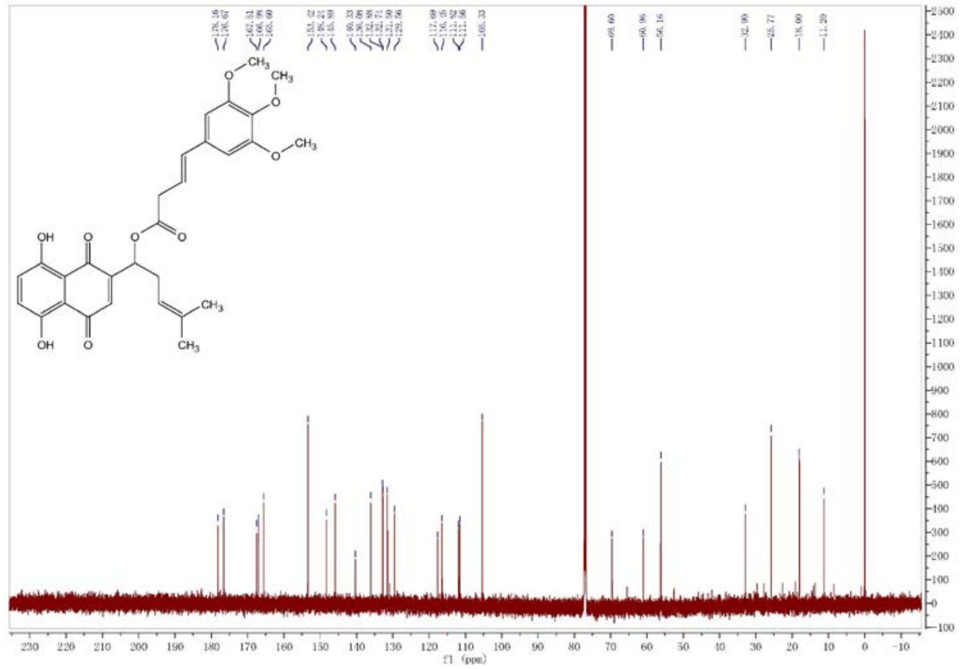

^{13}C NMR spectrum

1-(5,8-dihydroxy-1,4-dioxo-1,4-dihydronaphthalen-2-yl)-4-methylpent-3-en-1-yl-2-(4-chlorophenoxy)acetate (6)

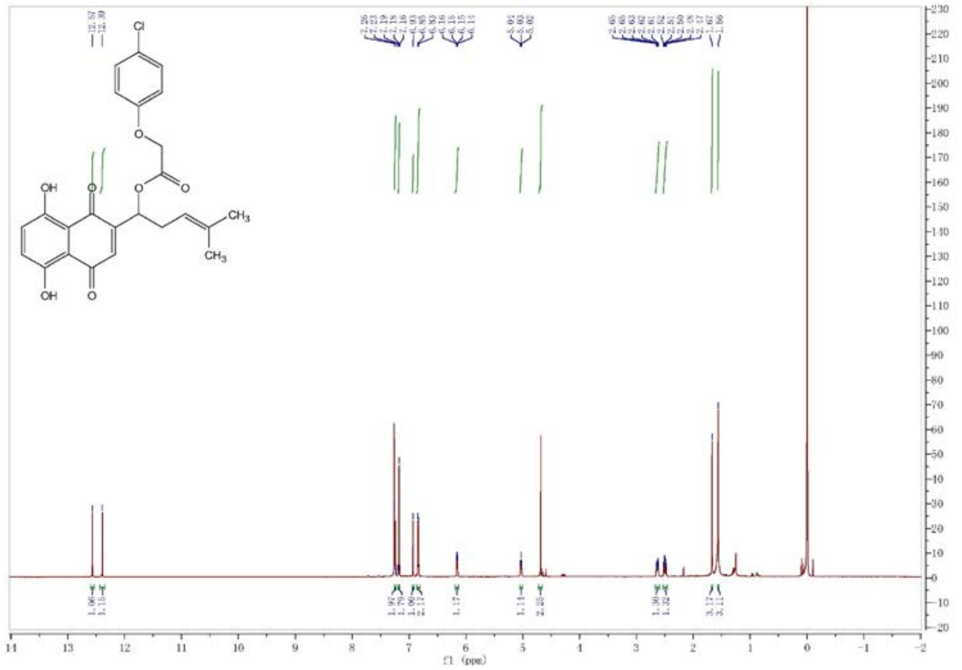

^{1}H NMR spectrum

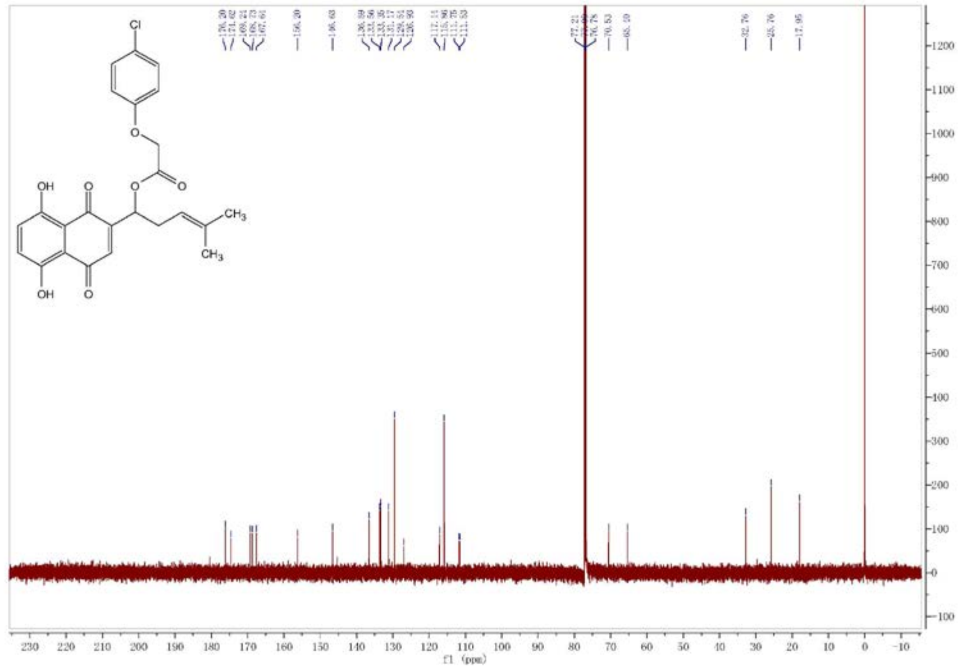

^{13}C NMR spectrum

1-(5,8-dihydroxy-1,4-dioxo-1,4-dihydronaphthalen-2-yl)-4-methylpent-3-en-1-yl-4-oxo-4-(piperidin-1-yl)butanoate (7)

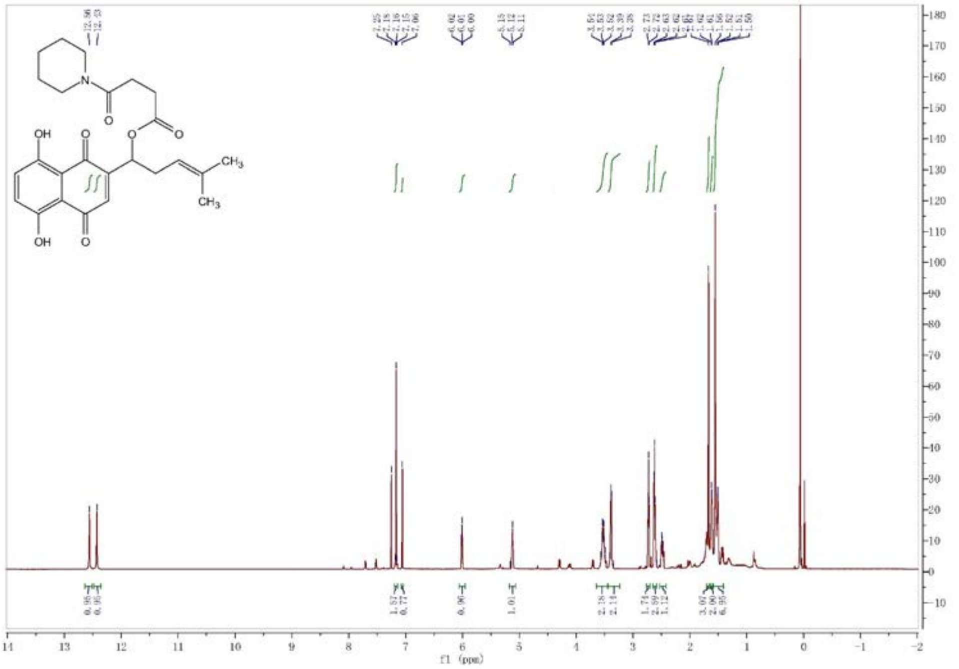

^1H NMR spectrum

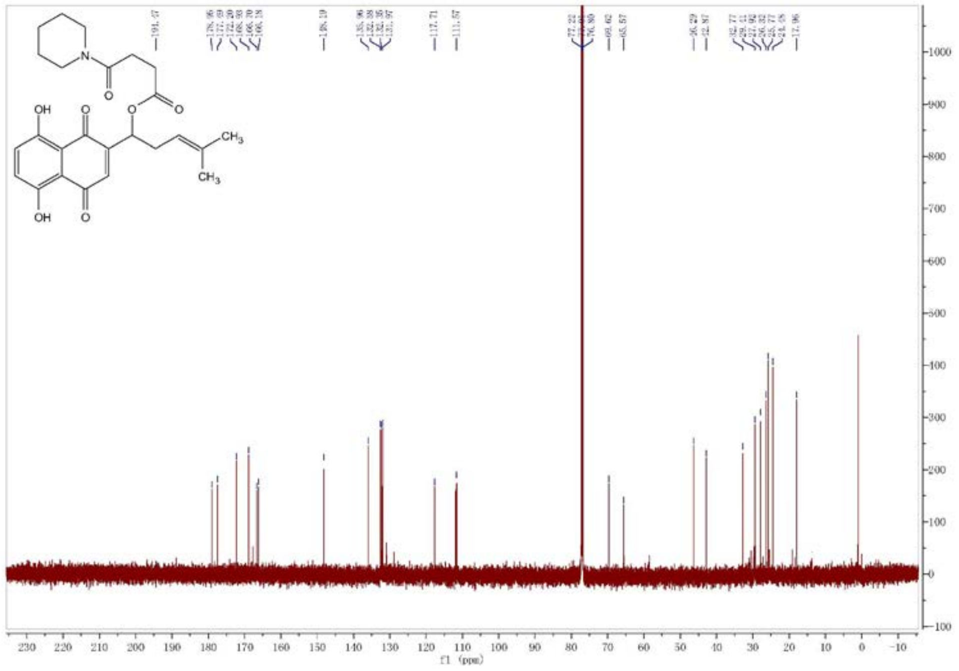

^{13}C NMR spectrum

1-(5,8-dihydroxy-1,4-dioxo-1,4-dihydronaphthalen-2-yl)-4-methylpent-3-en-1-yl 4-morpholino -4-oxobutanoate (8)

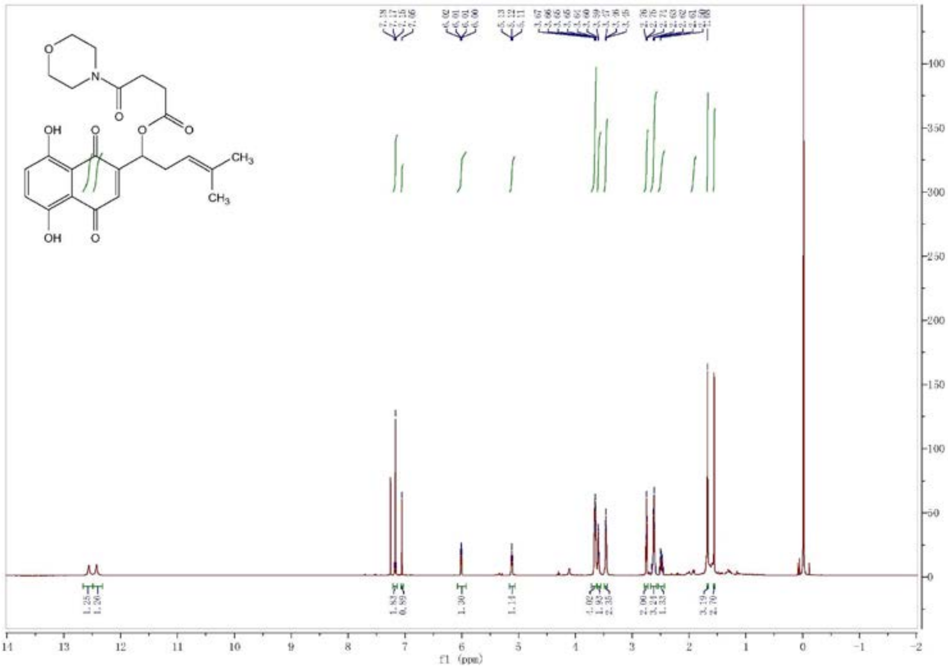

^{1}H NMR spectrum

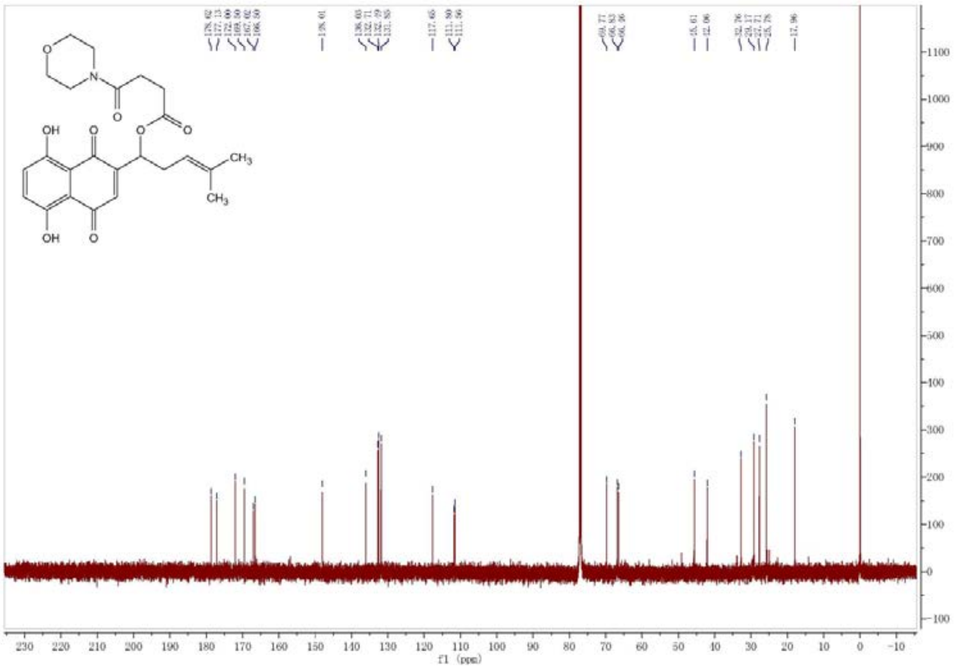

^{13}C NMR spectrum

1-(5,8-dihydroxy-1,4-dioxo-1,4-dihydronaphthalen-2-yl)-4-methylpent-3-en-1-yl-4-(3,4-dihydroquinolin-1(2H)-yl)-4-oxobutanoate (9)

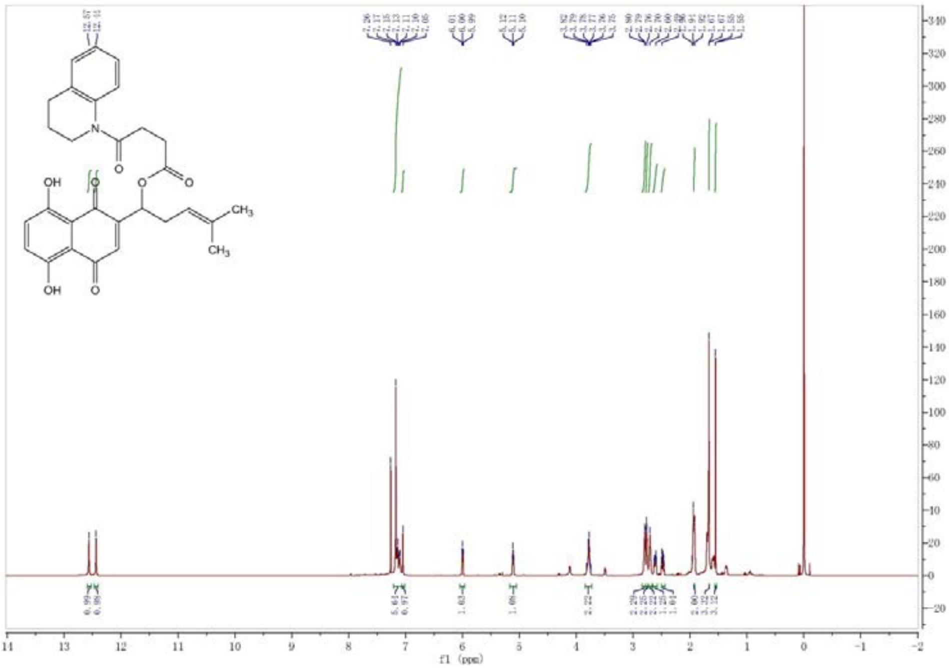

^{1}H NMR spectrum

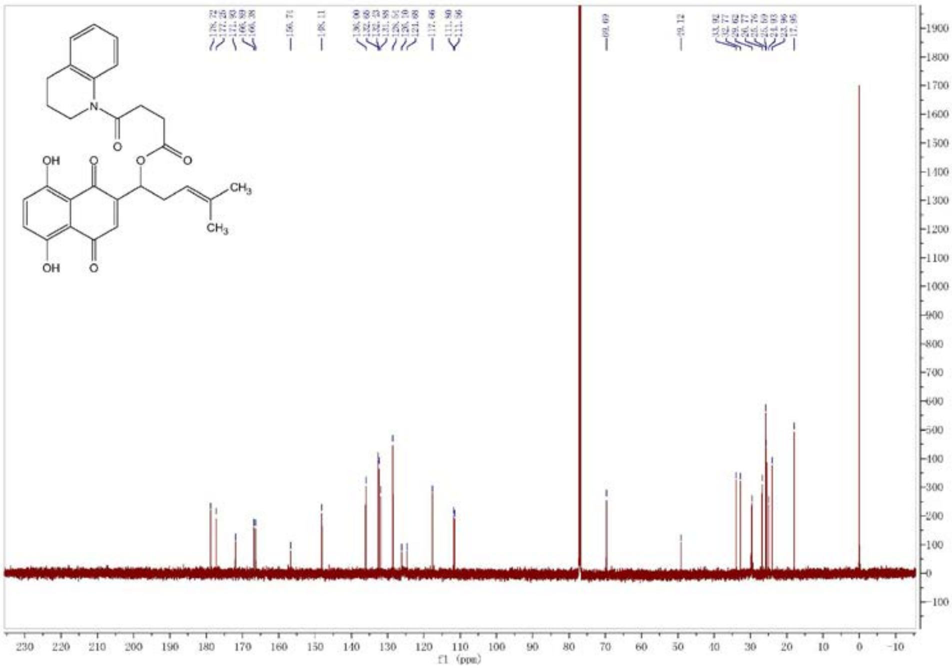

^{13}C NMR spectrum

1-(5,8-dihydroxy-1,4-dioxo-1,4-dihydronaphthalen-2-yl)-4-methylpent-3-en-1-yl-4-((4-methoxyphenyl)amino)-4-oxobutanoate (10)

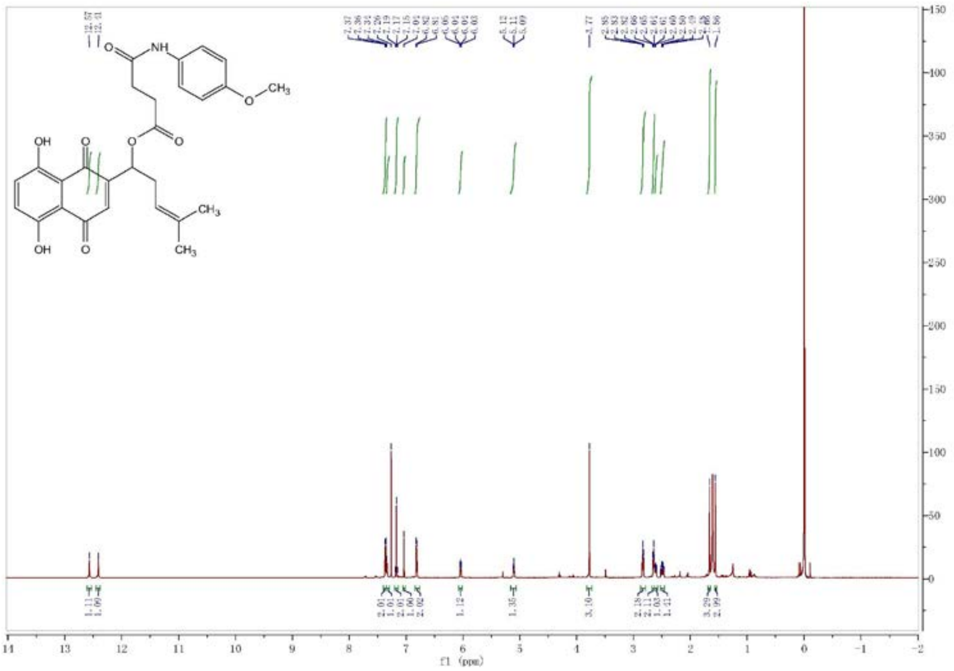

^{1}H NMR spectrum

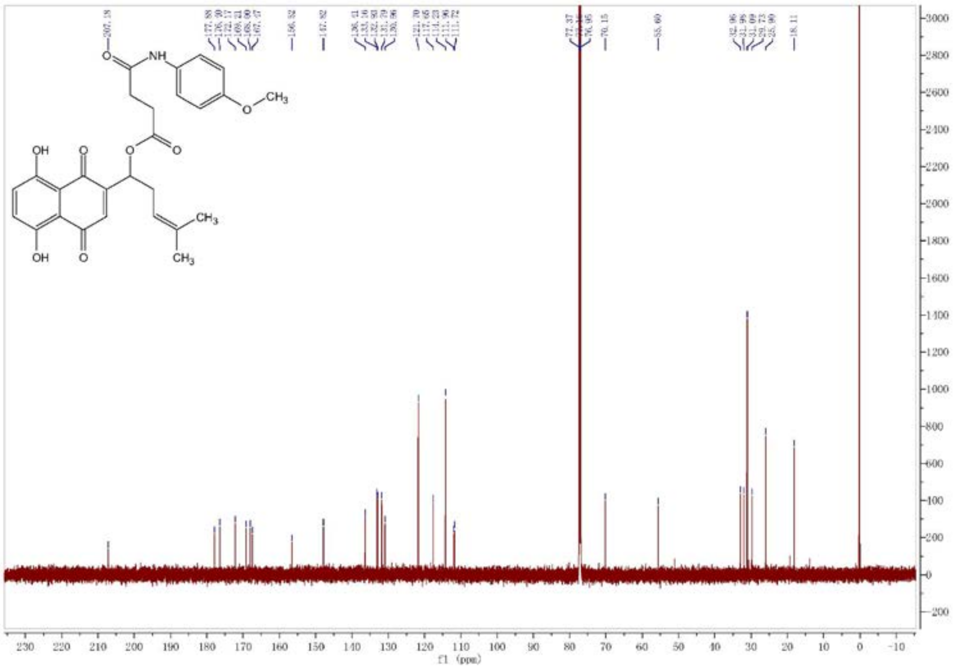

^{13}C NMR spectrum

1-(5,8-dihydroxy-1,4-dioxo-1,4-dihydronaphthalen-2-yl)-4-methylpent-3-en-1-yl 4-((4-fluoro-3-(trifluoromethyl)phenyl)amino)-4-oxobutanoate (11)

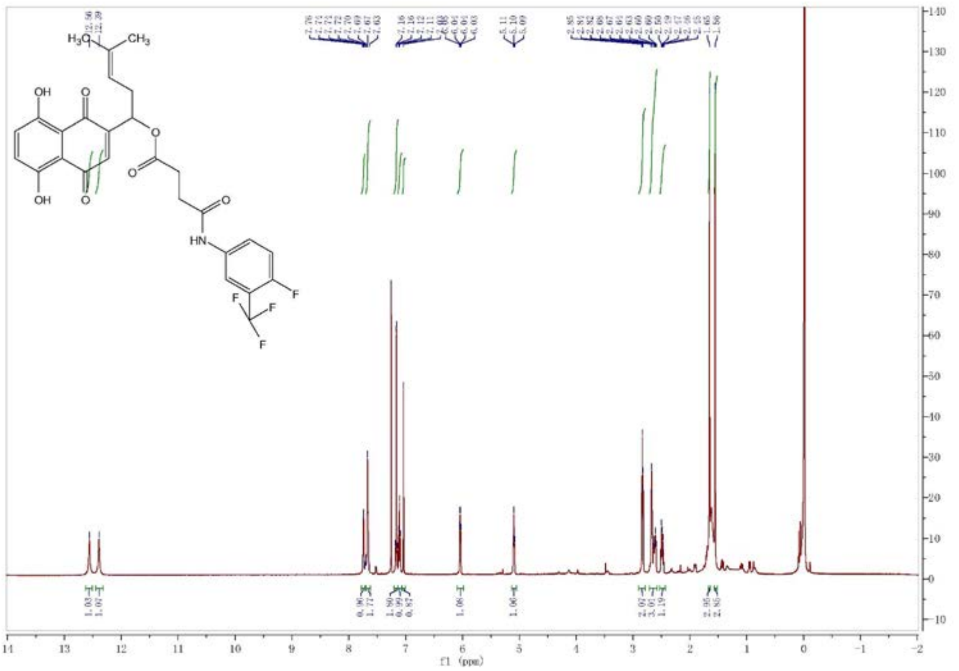

^{1}H NMR spectrum

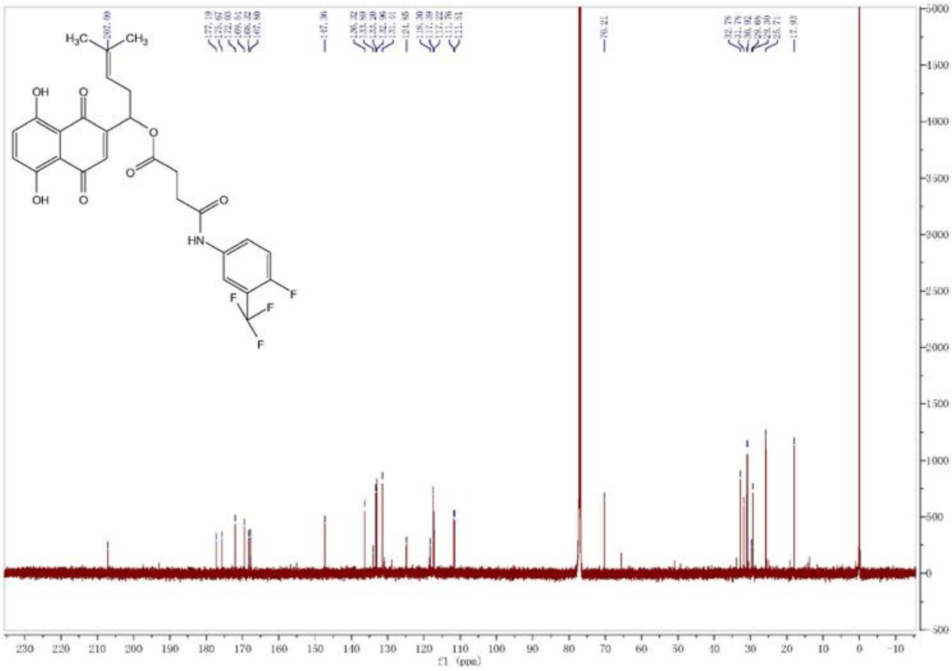

^{13}C NMR spectrum

1-(5,8-dihydroxy-1,4-dioxo-1,4-dihydronaphthalen-2-yl)-4-methylpent-3-en-1-yl-4-oxo-4-((4-phenoxyphenyl)amino)butanoate (12)

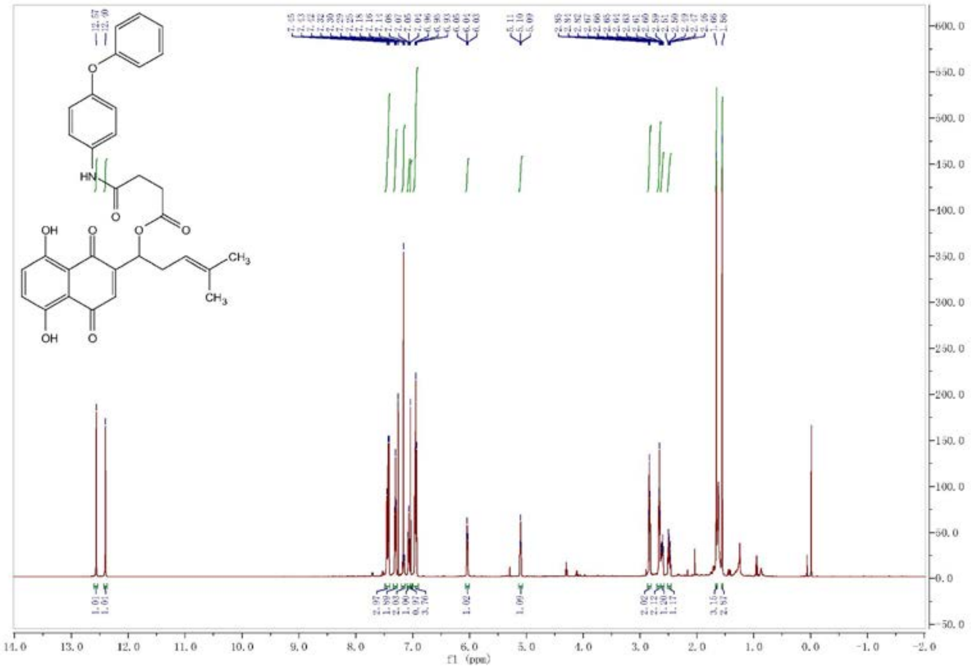

^{1}H NMR spectrum

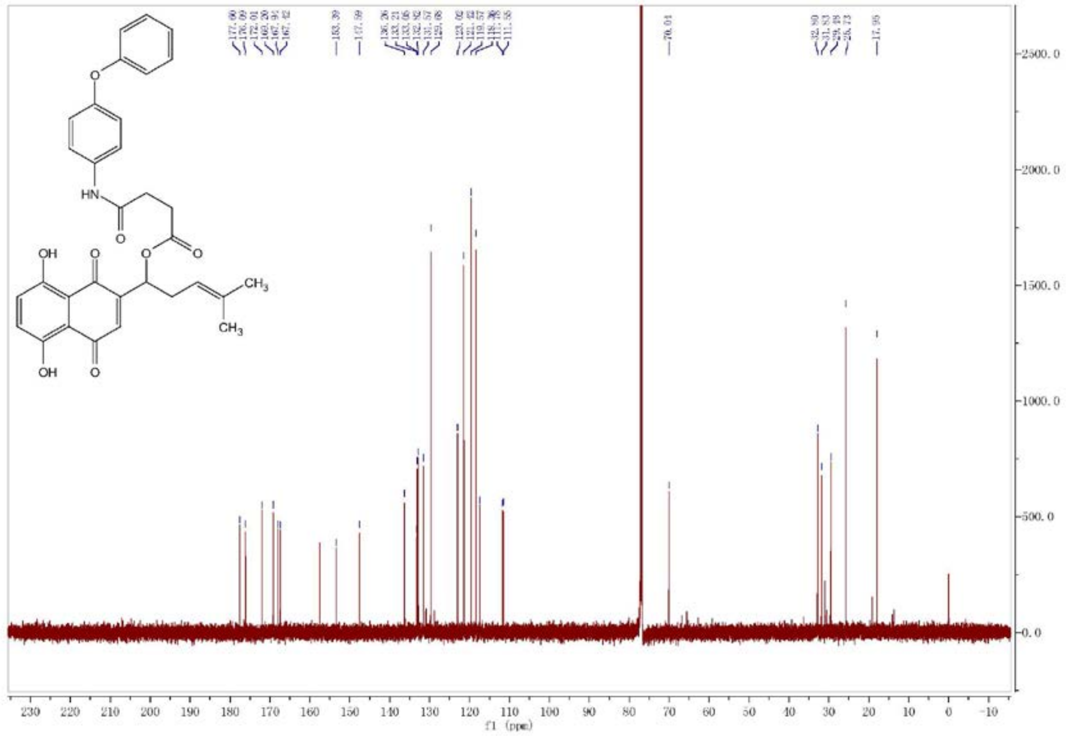

^{13}C NMR spectrum

1-(5,8-dihydroxy-1,4-dioxo-1,4-dihydronaphthalen-2-yl)-4-methylpent-3-en-1-yl-4-oxo-4-((4-(trifluoromethoxy)phenyl)amino)butanoate (13)

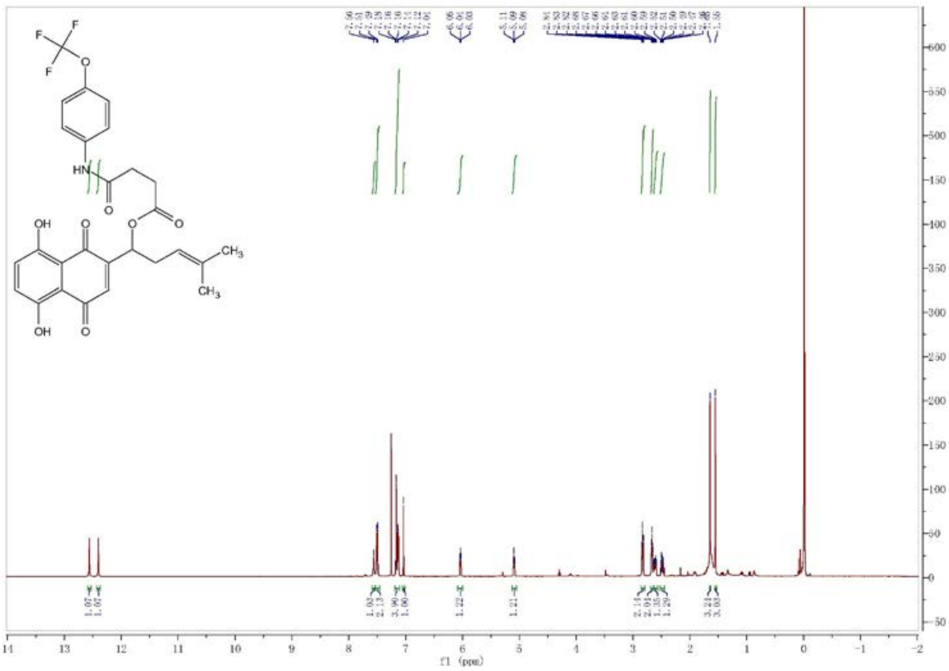

^{1}H NMR spectrum

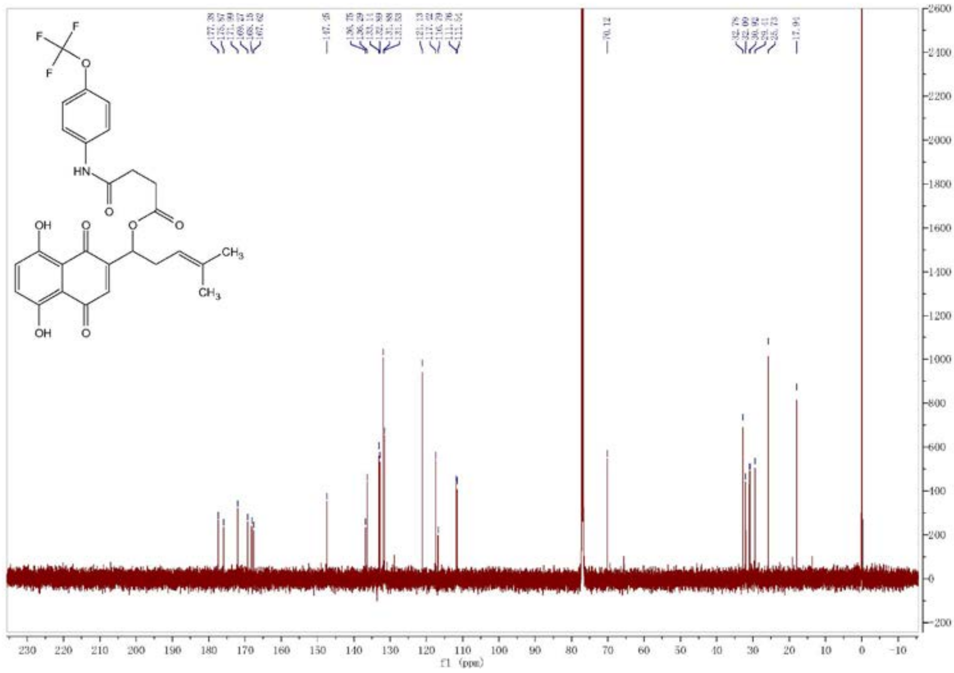

^{13}C NMR spectrum

1-(5,8-dihydroxy-1,4-dioxo-1,4-dihydronaphthalen-2-yl)-4-methylpent-3-en-1-yl-4-((4-bromophenyl)amino)-4-oxobutanoate (14)

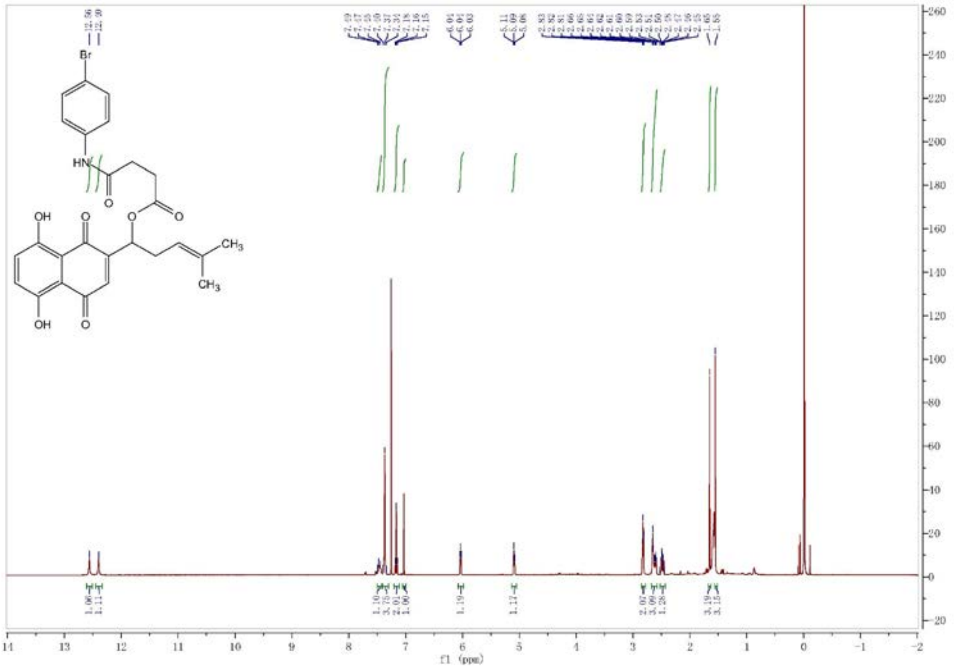

^{1}H NMR spectrum

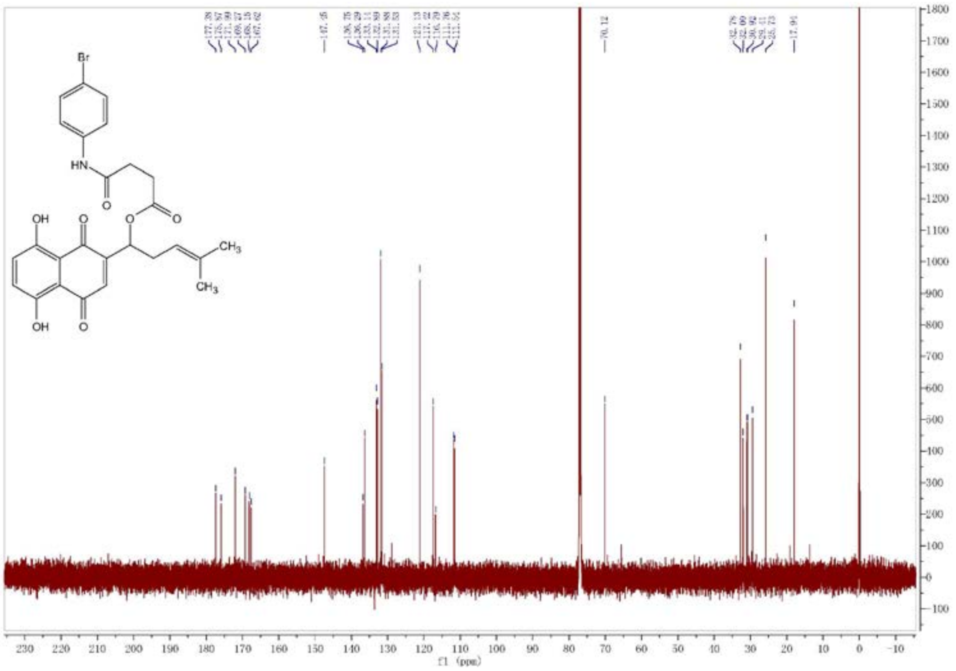

^{13}C NMR spectrum

1-(5,8-dihydroxy-1,4-dioxo-1,4-dihydronaphthalen-2-yl)-4-methylpent-3-en-1-yl-4-((2-methoxyphenyl)amino)-4-oxobutanoate (15)

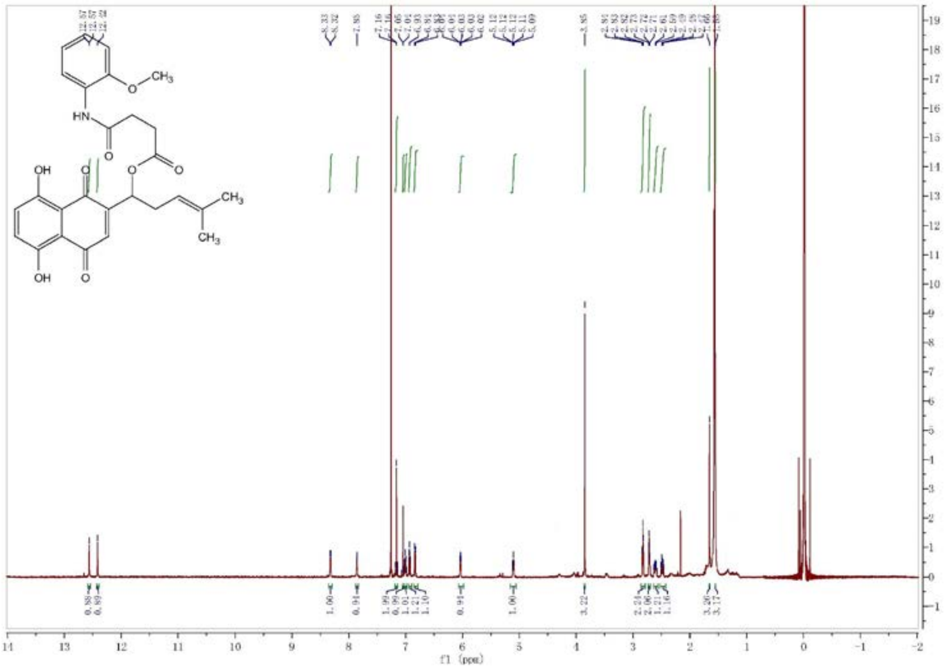

^{1}H NMR spectrum

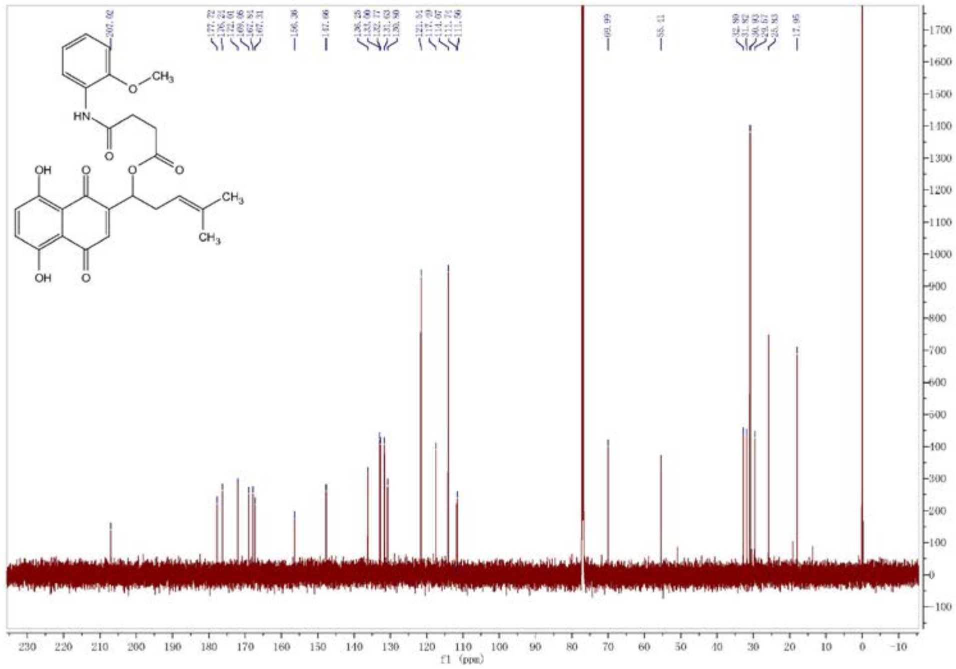

^{13}C NMR spectrum

1-(5,8-dihydroxy-1,4-dioxo-1,4-dihydronaphthalen-2-yl)-4-methylpent-3-en-1-yl-4-(naphthalen-1-ylamino)-4-oxobutanoate (16)

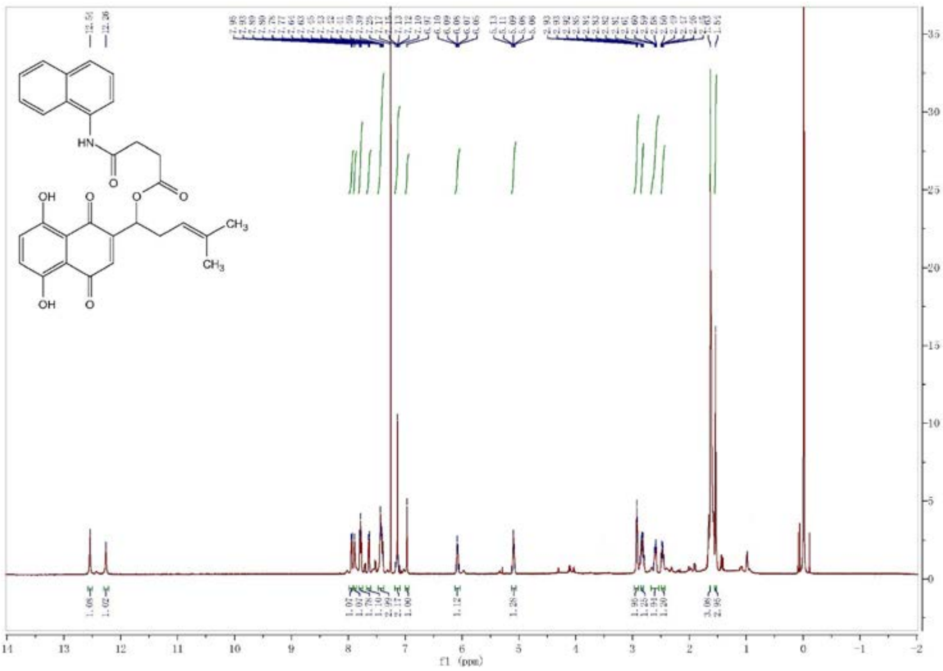

^{1}H NMR spectrum

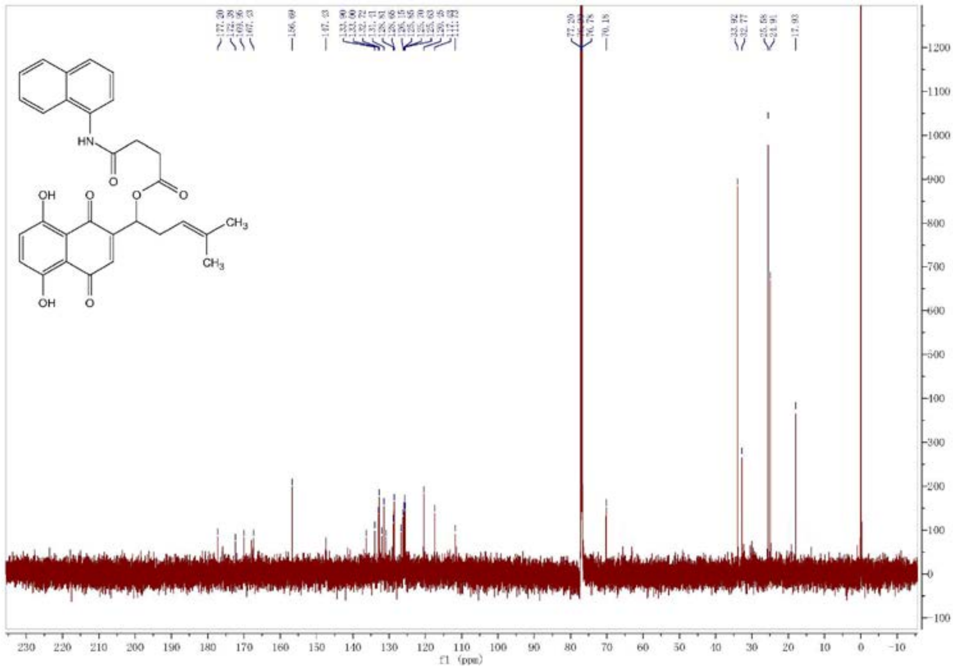

^{13}C NMR spectrum

1-(5,8-dihydroxy-1,4-dioxo-1,4-dihydronaphthalen-2-yl)-4-methylpent-3-en-1-yl-4-((3-chlorophenethyl)amino)-4-oxobutanoate (17)

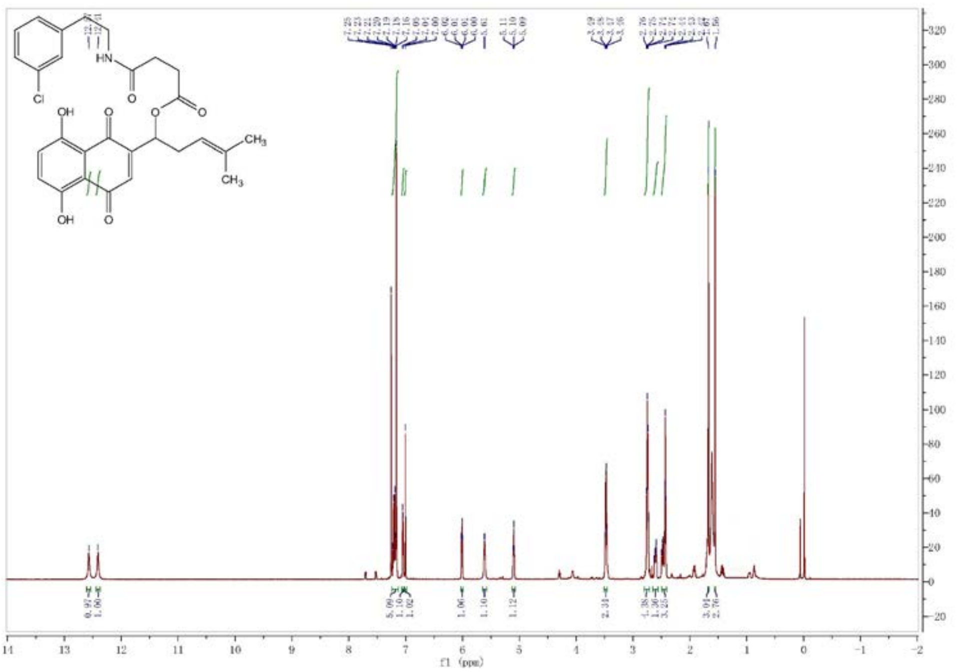

^{1}H NMR spectrum

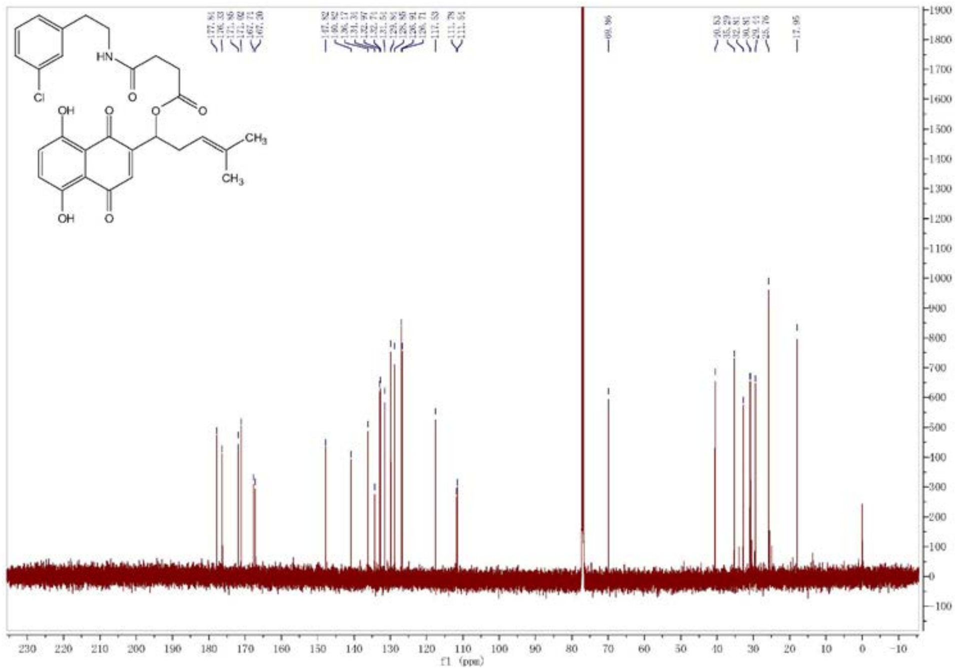

^{13}C NMR spectrum

1-(5,8-dihydroxy-1,4-dioxo-1,4-dihydronaphthalen-2-yl)-4-methylpent-3-en-1-yl-4-((2-chlorophenethyl)amino)-4-oxobutanoate (18)

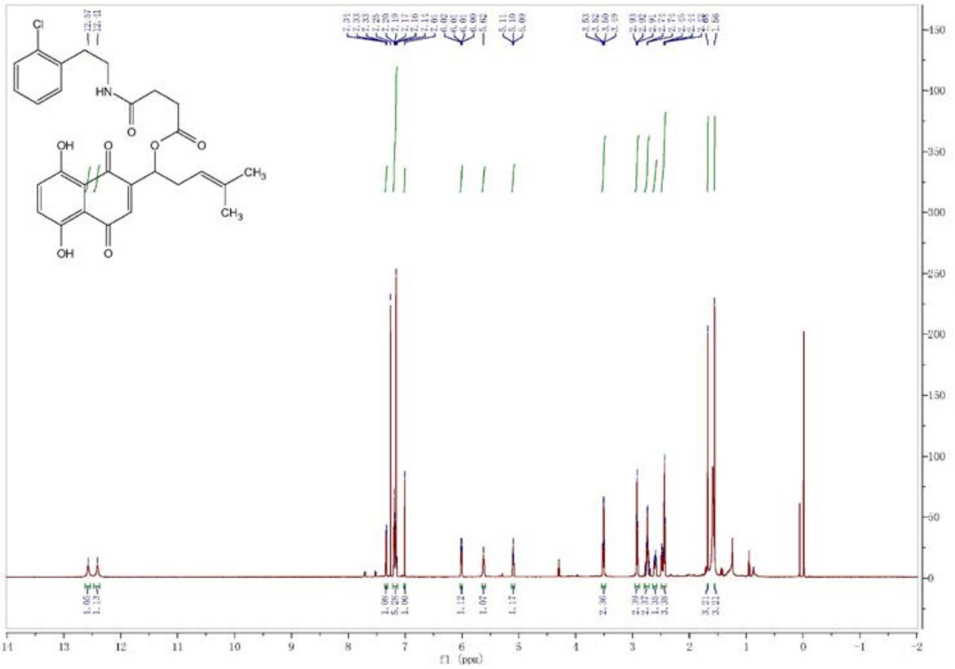

^{1}H NMR spectrum

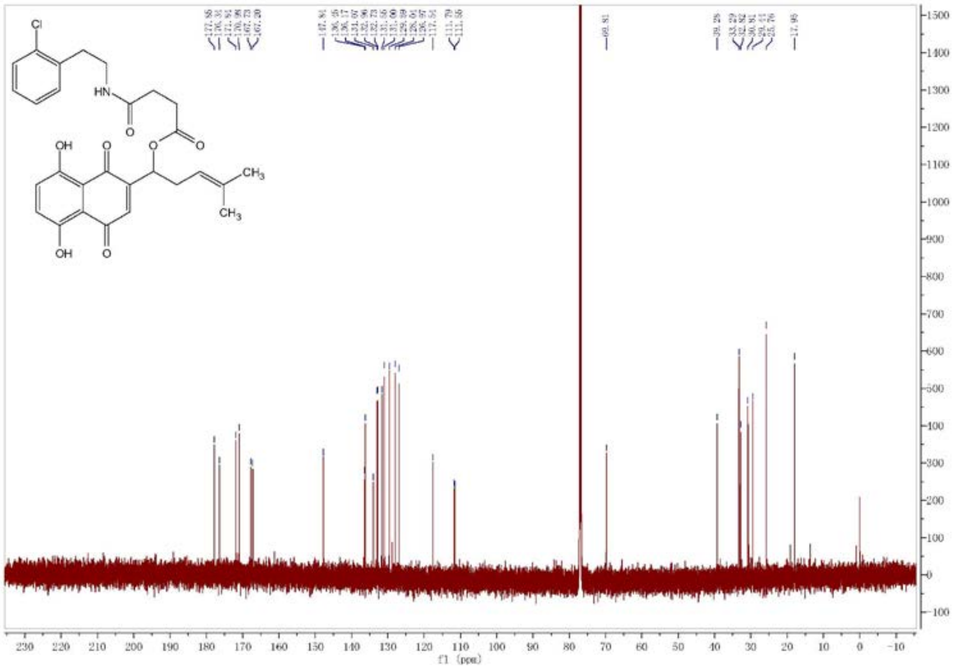

^{13}C NMR spectrum

1-(5,8-dihydroxy-1,4-dioxo-1,4-dihydronaphthalen-2-yl)-4-methylpent-3-en-1-yl-4-((4-bromophenethyl)amino)-4-oxobutanoate (19)

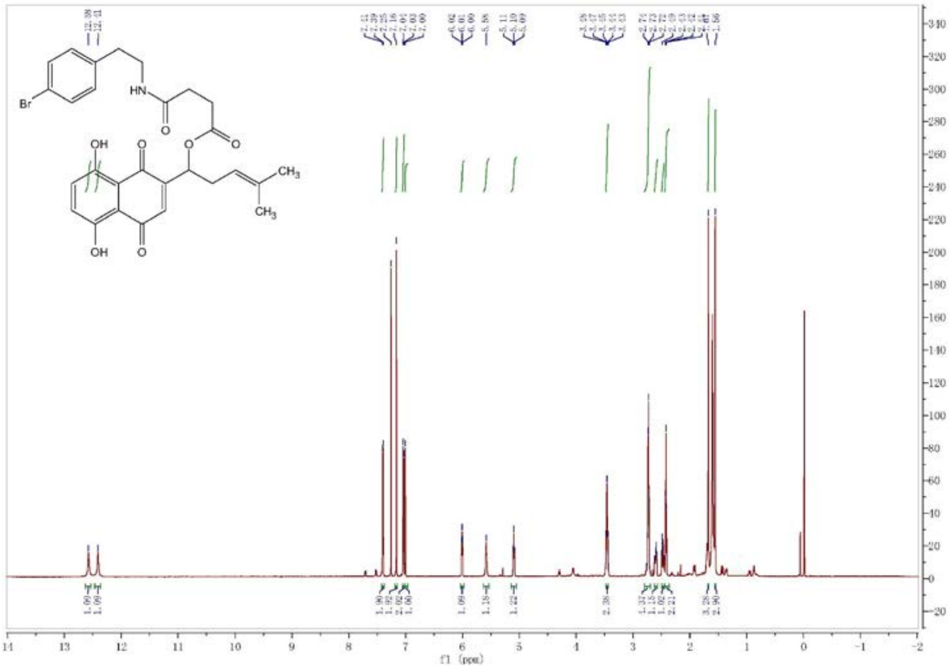

^{1}H NMR spectrum

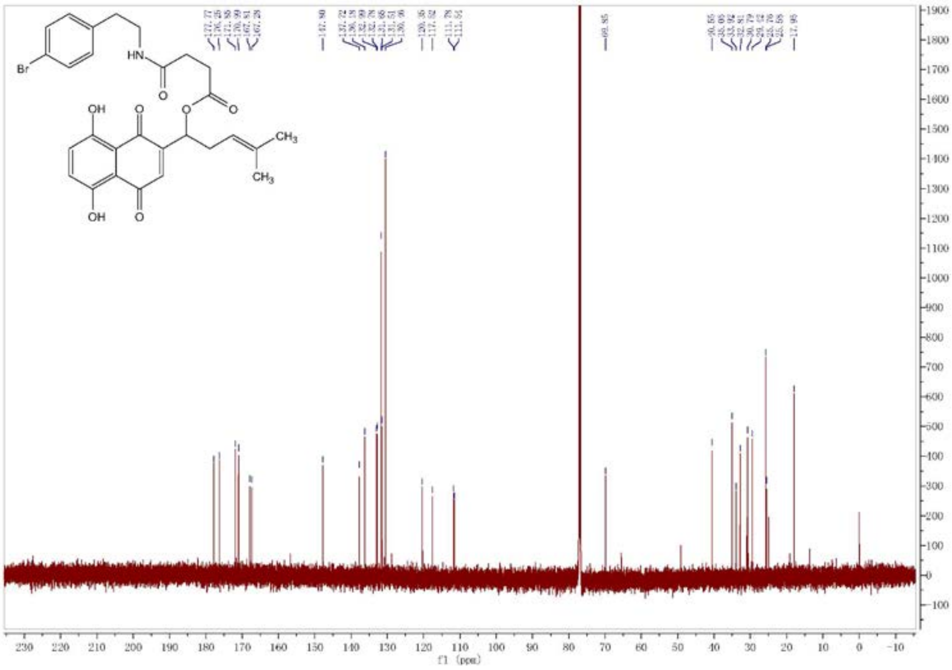

^{13}C NMR spectrum

1-(5,8-dihydroxy-1,4-dioxo-1,4-dihydronaphthalen-2-yl)-4-methylpent-3-en-1-yl 4-((4-fluorophenethyl)amino)-4-oxobutanoate (20)

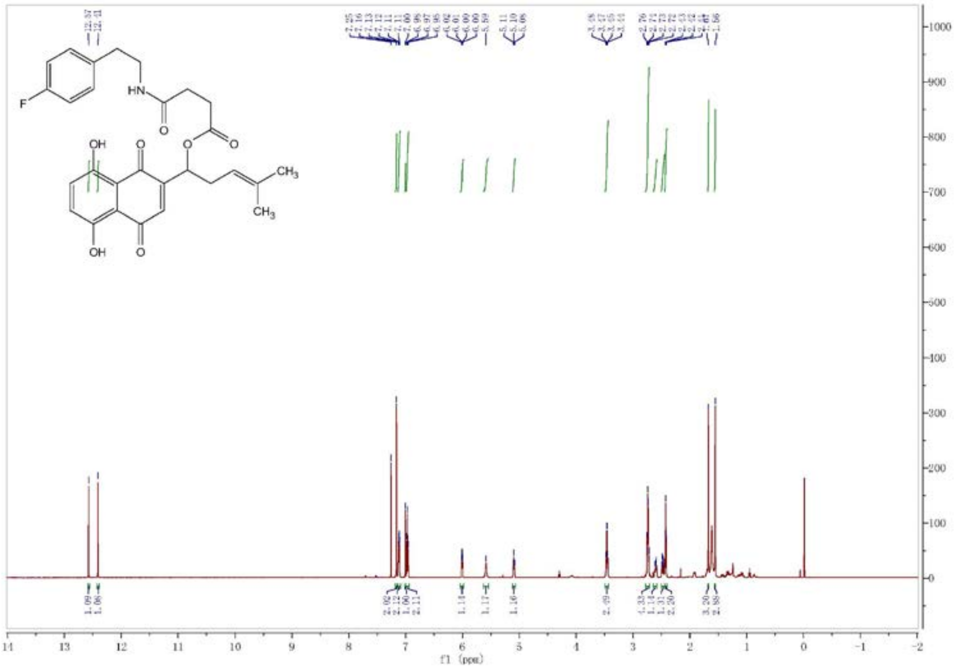

^{1}H NMR spectrum

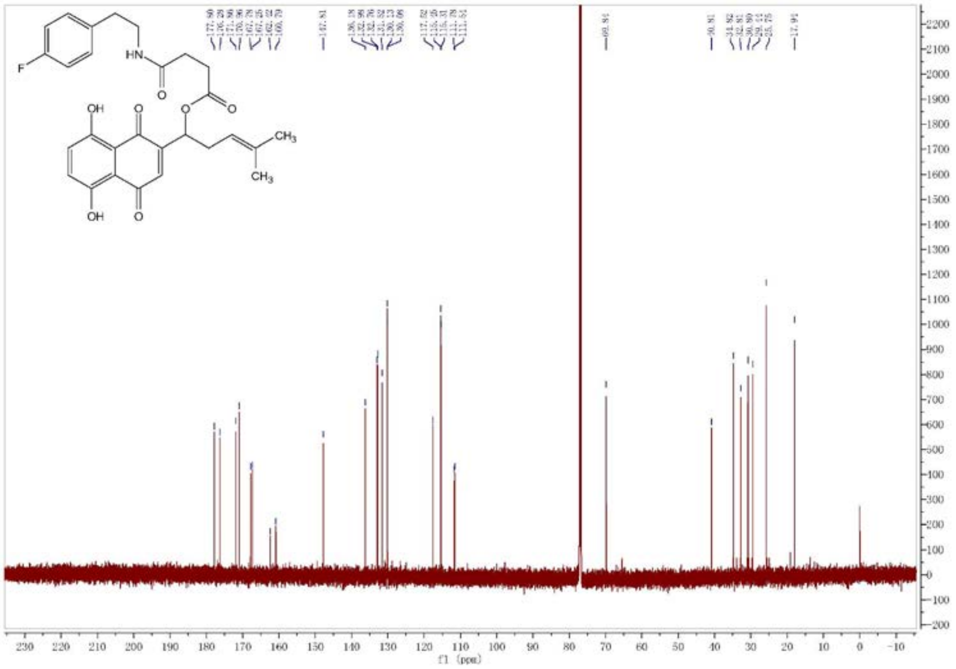

^{13}C NMR spectrum

1-(5,8-dihydroxy-1,4-dioxo-1,4-dihydronaphthalen-2-yl)-4-methylpent-3-en-1-yl 4-((2-fluorophenethyl)amino)-4-oxobutanoate (21)

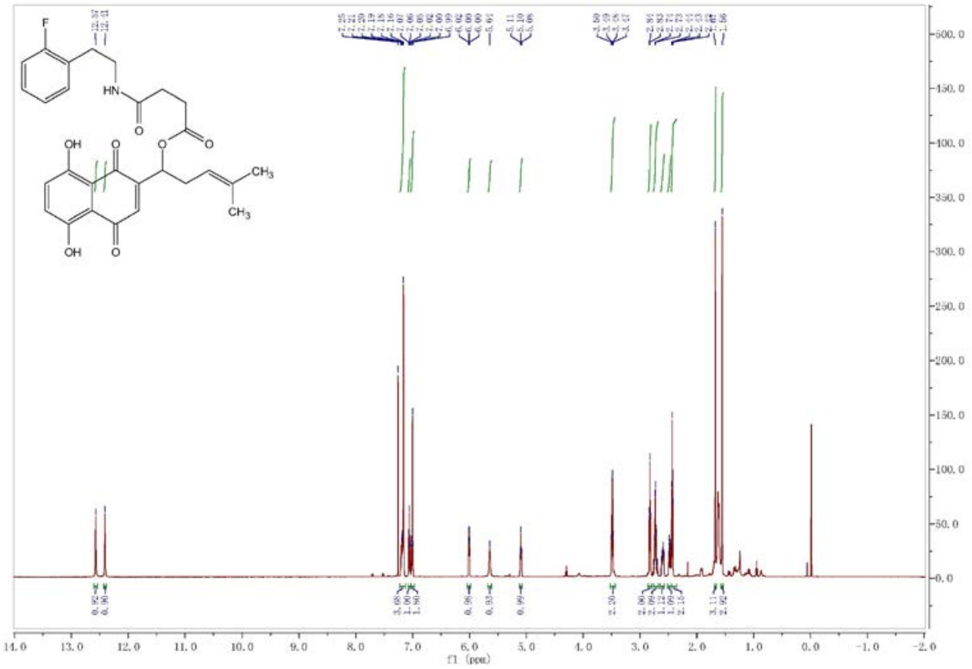

^{1}H NMR spectrum

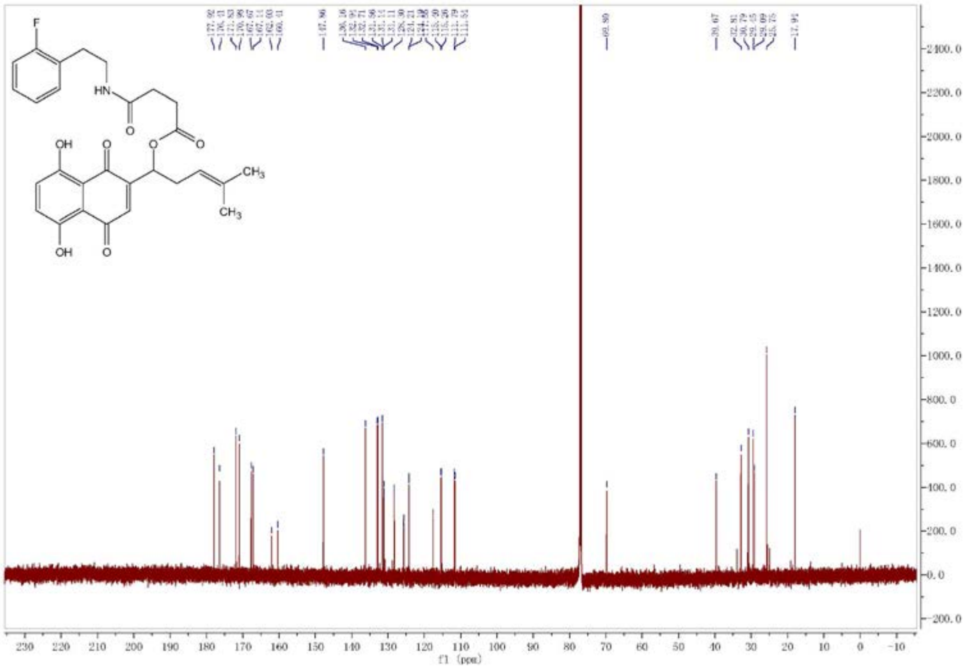

^{13}C NMR spectrum

1-(5,8-dihydroxy-1,4-dioxo-1,4-dihydronaphthalen-2-yl)-4-methylpent-3-en-1-yl 4-(diethylamino)-4-oxobutanoate (22)

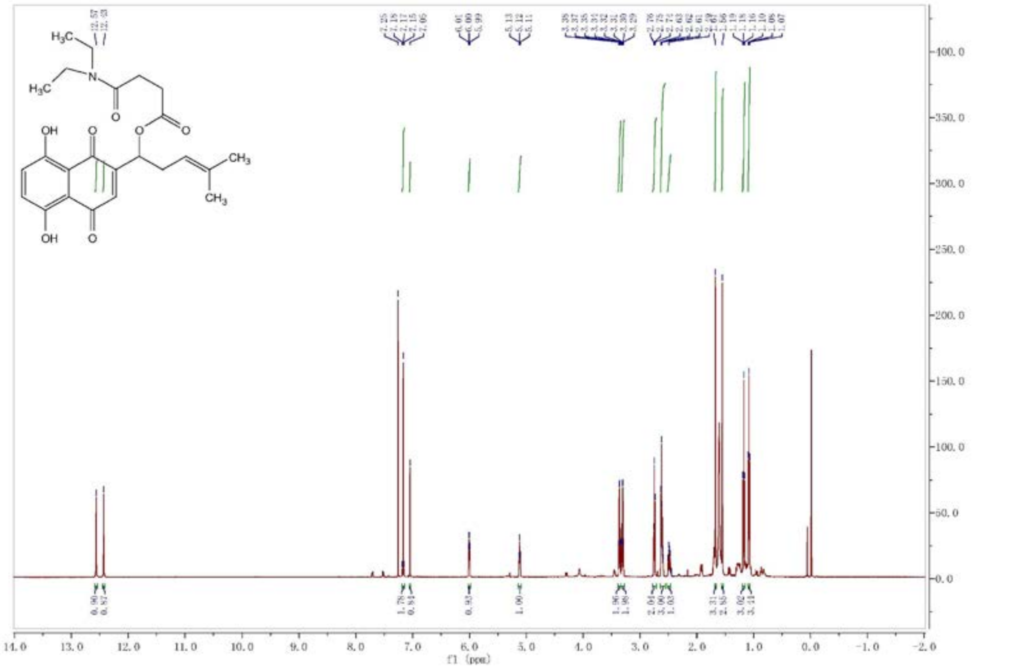

^{1}H NMR spectrum

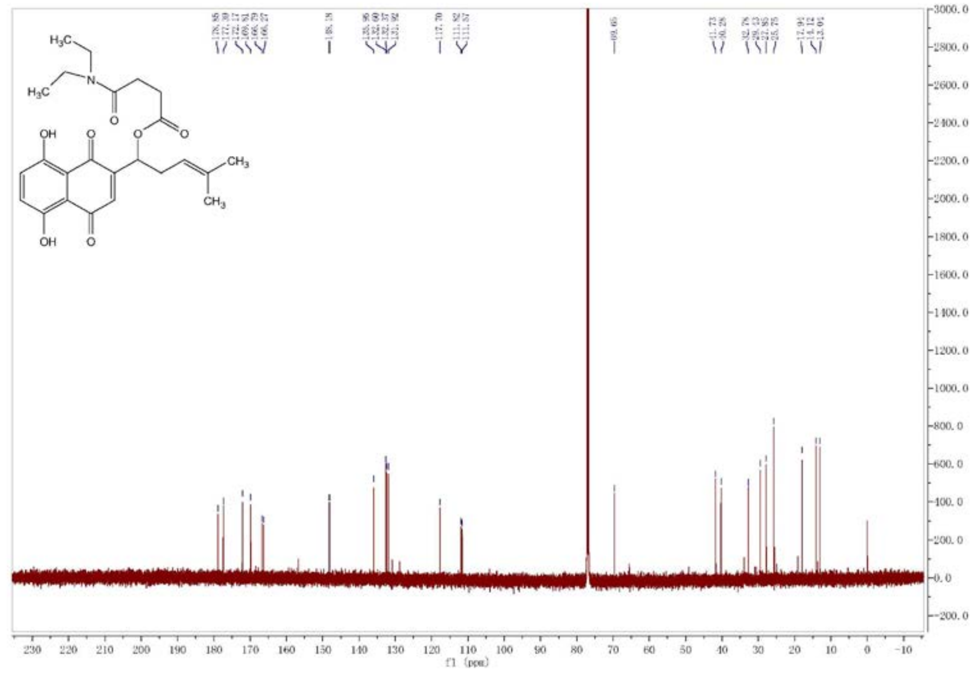

^{13}C NMR spectrum

1-(5,8-dihydroxy-1,4-dioxo-1,4-dihydronaphthalen-2-yl)-4-methylpent-3-en-1-yl 4-(dihexylamino)-4-oxobutanoate1-(5,8-dihydroxy-1,4-dioxo-1,4-dihydronaphthalen-2-yl)-4-methylpent-3-en-1-yl 4-(dihexylamino)-4-oxobutanoate (23)

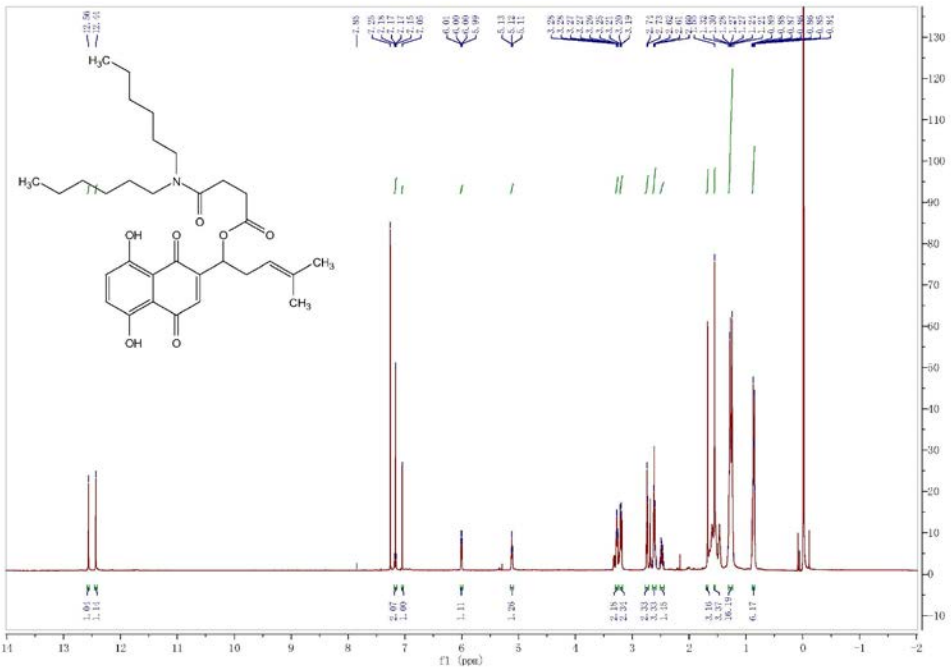

^{1}H NMR spectrum

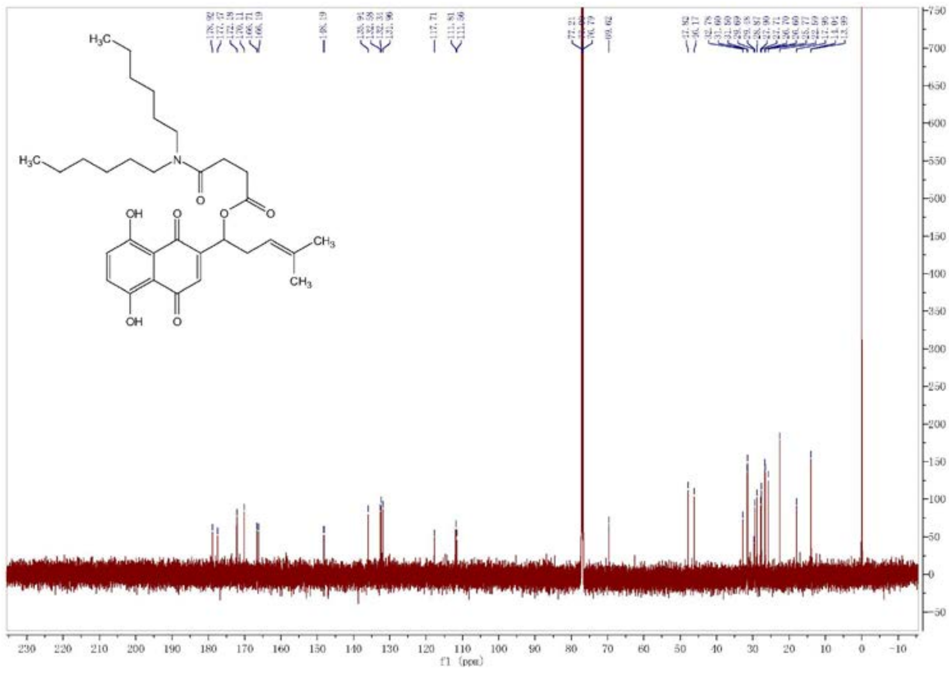

^{13}C NMR spectrum

1-(5,8-dihydroxy-1,4-dioxo-1,4-dihydronaphthalen-2-yl)-4-methylpent-3-en-1-yl (Z)-4-morpholino-4-oxobut-2-enoate (24)

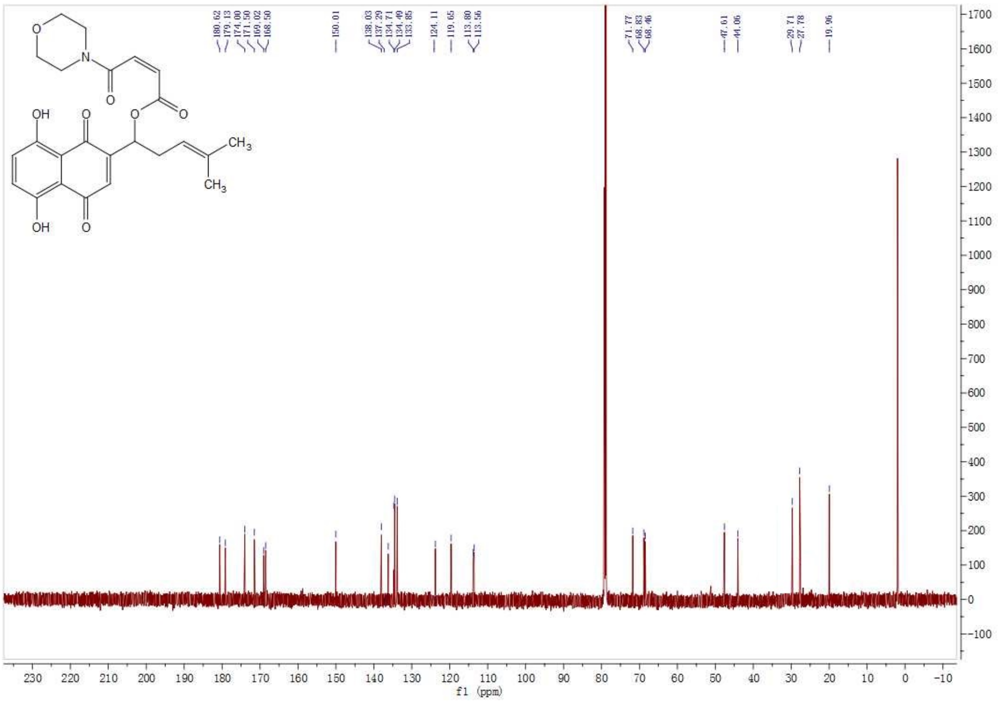

^{1}H NMR spectrum

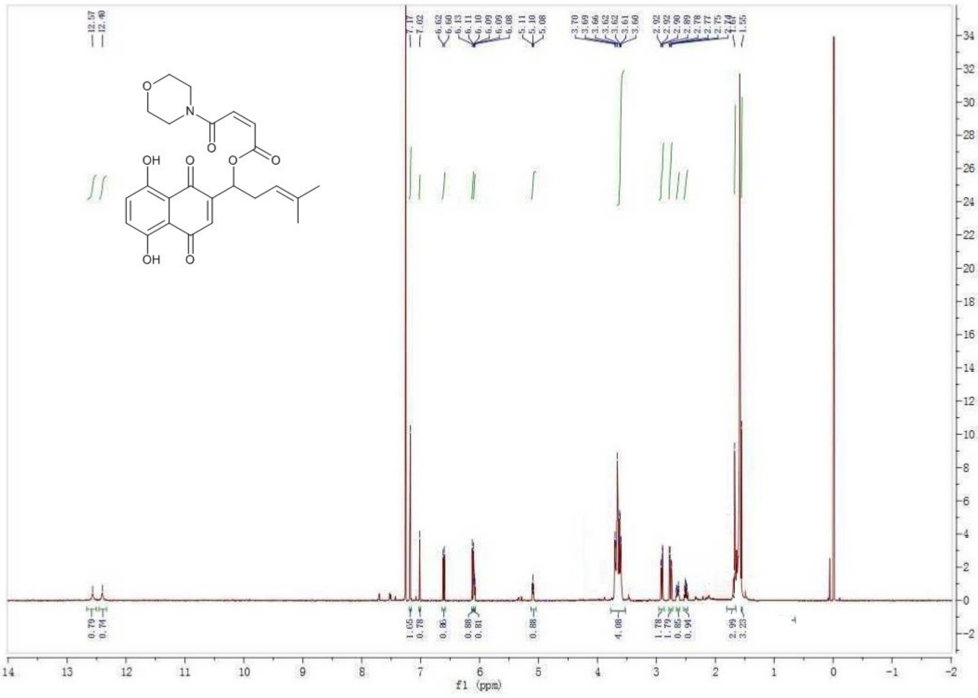

^{13}C NMR spectrum
